# Supplementary material for: Experimental and Computational Studies on the Interaction of DNA with Hesperetin Schiff Base CuII Complexes
Source: Int J Mol Sci. 2024 May 13;25(10):5283. doi: 10.3390/ijms25105283 (PMC11121494; doi:10.3390/ijms25105283)
Supplement: Supplementary file 1 [file ijms-25-05283-s001.zip › ijms-2986436-supplementary.pdf]

Section S1. $[Cu(L^1H_2)(AcO)]_{aq} + (H_2O)_{8\ aq} \rightleftarrows [Cu(L^1H_2)(H_2O)]^+_{aq} + [(H_2O)_7{\cdot}AcO]^-_{aq}$

This is Equation (2) of the main text.
In the next tables, for each molecule BS1 refers to the basis set 6-31G(d,p), applied to the main group elements, and the SDD plus f-functions, employed for the metal; BS2 refers to the basis set def2-TZVP for main group and def2-QZVP for d-block elements (see section 3.3 of the main text).

Table S1.1. $[Cu(L^1H_2)(AcO)]_{aq}$

Electronic Energy, BS1 (a.u.)	-1874.7512616
Thermal and entropic correction, BS1 (a.u.)	0.368166
Electronic Energy, BS2 (a.u.)	-3318.66519038

C	-2.349435	-0.906193	-0.547855
C	-1.288851	0.008753	0.053409
H	-2.082579	-1.123840	-1.589690
H	-1.592981	0.329890	1.058177
H	-1.225338	0.900918	-0.576922
C	-1.165477	-2.812785	0.238845
C	-1.247791	-4.181914	0.396313
C	-0.058075	-4.923069	0.487406
C	1.182806	-4.304862	0.409134
C	1.291910	-2.906043	0.254904
C	0.082591	-2.112798	0.176164
H	-2.213148	-4.671184	0.434996
H	2.096390	-4.888184	0.466871
C	0.052834	-0.677287	0.142279
N	1.157411	0.029072	0.231863
O	-2.365368	-2.156342	0.177882
O	-0.195781	-6.271515	0.638493
H	0.679749	-6.687543	0.665051
C	-3.733476	-0.314391	-0.485647

C	-4.371205	-0.154258	0.755207
C	-4.376891	0.107501	-1.647826
C	-5.633825	0.416549	0.823267
H	-3.894529	-0.480954	1.673854
C	-5.647841	0.694860	-1.587673
H	-3.892056	-0.017624	-2.611351
C	-6.280061	0.850183	-0.356386
H	-6.135266	1.018199	-2.499530
O	-6.247801	0.557599	2.043975
H	-7.113448	0.972204	1.895334
O	-7.519630	1.398492	-0.150736
C	-8.243540	1.865736	-1.293756
H	-8.446213	1.047052	-1.993375
H	-9.185163	2.260559	-0.910568
H	-7.694184	2.661444	-1.809400
O	2.506970	-2.411795	0.188791
N	1.086360	1.410006	0.275435
H	0.222724	1.873149	0.535534
C	2.252582	2.084383	0.246893
O	3.341897	1.446290	0.167409
C	2.221598	3.558087	0.310902
C	3.362165	4.221694	0.790947
C	1.104813	4.298895	-0.111778
C	3.376405	5.612035	0.867346
H	4.221700	3.641968	1.109332
C	1.129299	5.690047	-0.036471
H	0.233153	3.802369	-0.526541
C	2.260578	6.347338	0.455788
H	4.255988	6.121943	1.247400
H	0.268956	6.260815	-0.371136
H	2.274772	7.431548	0.512620
C	5.499917	-0.916980	-1.045907
O	4.876956	-0.672779	-2.098683
O	4.950958	-0.971329	0.131184
C	6.994894	-1.173777	-1.062602
H	7.208695	-2.143749	-0.603412
H	7.502441	-0.410305	-0.464272
H	7.381804	-1.154956	-2.082432
Cu	3.039345	-0.565395	0.162222

Table S1.2. $(H_2O)_{8\ aq}$

Electronic Energy, BS1 (a.u.)	-611.57938466
Thermal and entropic correction, BS1 (a.u.)	0.158165
Electronic Energy, BS2 (a.u.)	-611.881953741

H	-0.593263	-1.414510	1.478025
O	0.256970	-1.907593	1.425579
H	0.927771	-1.191026	1.499261
O	1.890460	0.294749	1.298777
H	2.023340	0.303247	0.309006
H	2.762621	0.425627	1.694661
H	0.439771	-2.808839	-1.613773
O	0.304608	-1.925398	-1.245189
H	0.302346	-2.029701	-0.251909
H	1.461722	-0.583304	-1.440704
O	1.951763	0.267704	-1.372045
H	1.236206	0.937353	-1.463483
O	-1.935533	-0.266038	1.242506
H	-2.822271	-0.388086	1.607549
H	-2.032814	-0.294100	0.248899
O	-0.306082	1.941284	1.374905
H	0.540980	1.448009	1.465221
H	-0.980329	1.227199	1.441230
O	-0.259425	1.895629	-1.296566
H	-0.385433	2.772206	-1.684249
H	-0.292051	2.019287	-0.306189
O	-1.902194	-0.300479	-1.428681
H	-1.409604	0.549000	-1.498475
H	-1.183534	-0.971224	-1.479367

Table S1.3. $[Cu(L^1H_2)(H_2O)]^+_{aq}$

Electronic Energy, BS1 (a.u.)	=	-1722.54133998
Thermal and entropic correction, BS1 (a.u.)	=	0.348554

Electronic Energy, BS2 (a.u.) = -3166.3895989

C	-1.985048	-0.764904	-0.537187
C	-0.824528	0.016043	0.069672
H	-1.766946	-0.958514	-1.595102
H	-1.052015	0.281225	1.110235
H	-0.709834	0.945069	-0.495746
C	-0.961745	-2.806362	0.126029
C	-1.160952	-4.165793	0.265491
C	-0.041314	-5.013698	0.273803
C	1.245120	-4.510675	0.118821
C	1.472205	-3.127580	-0.021876
C	0.341750	-2.223994	0.006506
H	-2.163535	-4.565748	0.353178
H	2.100978	-5.178016	0.103225
C	0.447605	-0.793338	0.031352
N	1.619325	-0.200698	0.064874
O	-2.095021	-2.040312	0.135126
O	-0.289169	-6.345689	0.419757
H	0.547245	-6.837151	0.406445
C	-3.302781	-0.046774	-0.410981
C	-3.926650	0.069361	0.841380
C	-3.888822	0.545751	-1.528478
C	-5.121060	0.765140	0.964322
H	-3.493917	-0.389136	1.724627
C	-5.087091	1.262039	-1.411035
H	-3.414885	0.455739	-2.501297
C	-5.706588	1.374526	-0.168566
H	-5.528926	1.718621	-2.288443
O	-5.725575	0.857144	2.194273
H	-6.549399	1.359784	2.085350
O	-6.877962	2.038055	0.090001
C	-7.542757	2.677853	-1.004327
H	-7.830338	1.949669	-1.771003
H	-8.437402	3.137733	-0.583033
H	-6.908909	3.451229	-1.452678
O	2.722753	-2.746554	-0.177244
N	1.700685	1.176600	0.155740
H	0.921124	1.711175	0.522112
C	2.919474	1.725170	-0.007303
O	3.911150	0.975607	-0.258818

C	3.072999	3.185995	0.118344
C	4.344847	3.687767	0.439625
C	2.000599	4.071300	-0.085857
C	4.536625	5.060279	0.574023
H	5.167246	2.997590	0.592494
C	2.202663	5.443350	0.045925
H	1.021225	3.702446	-0.373921
C	3.466771	5.938970	0.378888
H	5.518490	5.444511	0.831590
H	1.375419	6.125949	-0.119891
H	3.618872	7.009157	0.481142
Cu	3.403392	-0.971425	-0.233410
O	5.307539	-1.669030	-0.449191
H	5.747585	-1.200394	-1.176730
H	5.261835	-2.597273	-0.730628

Table S1.4. $[(H_2O)_7{\cdot}AcO]^{-}_{aq}$

Electronic Energy, BS1 (a.u.)	-764.238502286
Thermal and entropic correction, BS1 (a.u.)	0.187176
Electronic Energy, BS2 (a.u.)	-764.599379292

C	0.182621	-1.409710	0.531281
O	1.283094	-0.851867	0.830026
C	0.220791	-2.769310	-0.146177
H	0.121559	-3.543107	0.624049
H	-0.618966	-2.877615	-0.836112
H	1.166714	-2.923635	-0.668229
O	-0.954111	-0.913456	0.807391
H	1.427875	0.892693	1.042882
H	-1.440035	0.787293	0.951863
O	-1.878147	1.661643	0.841349
O	1.617684	1.857449	0.982773
H	1.054778	2.139812	0.227064
H	3.130239	1.588589	-0.028608
H	2.646527	-1.278772	-0.099496

O	3.432494	-1.447703	-0.680057
H	4.115188	-1.738864	-0.059357
O	3.883720	1.358830	-0.613492
H	3.787428	0.391204	-0.716686
H	-2.252653	-1.409041	-0.148390
O	-3.012204	-1.624812	-0.752965
H	-3.580128	-2.196372	-0.216991
H	-3.793297	-0.069549	-0.652780
O	-4.159596	0.837570	-0.498124
H	-4.750614	0.715717	0.258447
H	-0.867701	2.242622	-0.399814
O	-0.171845	2.548437	-1.042118
H	-0.186427	3.511643	-0.950066
H	-2.709660	1.432762	0.363327

Section S2. $[Cu(L^1H_2^{am})(H_2O)]^+_{aq} + (H_2O)_{14\,aq} \rightleftarrows [Cu(L^1H^{im})(H_2O)]_{aq} + [(H_2O)_{14}\cdot H]^+_{aq}$

This is Equation (3) of the main text.
In the next tables, for each molecule BS1 refers to the basis set 6-31G(d,p), applied to the main group elements, and the SDD plus f-functions, employed for the metal; BS2 refers to the basis set def2-TZVP for main group and def2-QZVP for d-block elements (see section 3.3 of the main text).

$[Cu(L^1H_2^{am})(H_2O)]^+_{aq}$. See $[Cu(L^1H_2)(H_2O)]^+_{aq}$ in Table S1.3.

Table S2.1. $(H_2O)_{14\,aq}$

Electronic Energy, BS1 (a.u.)	-1070.28441612
Thermal and entropic correction, BS1 (a.u.)	0.292603
Electronic Energy, BS2 (a.u.)	-1070.80705726

O	3.553290	0.181752	-1.254464
H	3.602846	0.072659	-0.276425
H	2.963691	0.963301	-1.356848
O	1.875248	2.362026	-1.219451
H	0.959044	2.028250	-1.382967
H	2.000857	3.111161	-1.817263
H	-1.493221	1.438825	-1.516241
O	-0.532889	1.217952	-1.499775
H	-0.494831	0.225146	-1.485199
H	2.651070	-1.178592	-1.539798
O	2.114905	-2.024041	-1.572611
H	2.561693	-2.609834	-2.198194
O	-0.493172	-1.453215	-1.253162
H	-0.476797	-1.467094	-0.264549
H	0.413949	-1.737432	-1.518175

H	0.440509	1.723172	1.490773
O	-0.435866	1.317289	1.259330
H	-0.450837	1.322279	0.272705
H	-0.436007	-0.367908	1.493742
O	-0.524753	-1.356608	1.489904
H	-1.495663	-1.516811	1.531446
H	2.506040	1.415361	1.607210
O	2.060169	2.289893	1.541325
H	2.069361	2.466567	0.574970
O	1.972047	-2.459142	1.148996
H	1.045644	-2.180409	1.315723
H	2.047146	-2.441970	0.167885
O	3.299720	-0.165719	1.457462
H	4.092009	-0.351348	1.979680
H	2.789890	-1.024541	1.418780
O	-3.213108	1.420921	-1.209834
H	-3.845332	2.061956	-1.561649
H	-3.268223	1.482381	-0.214359
O	-3.253078	-1.321475	-1.429378
H	-2.292094	-1.477829	-1.541100
H	-3.330100	-0.340852	-1.466810
O	-3.229109	-1.381691	1.250502
H	-3.892098	-1.987672	1.607978
H	-3.303173	-1.437219	0.256883
O	-3.177069	1.364891	1.462874
H	-3.291552	0.388149	1.504142
H	-2.204493	1.477641	1.543915

Table S2.2. $[Cu(L^1H^{im})(H_2O)]_{aq}$

Electronic Energy, BS1 (a.u.)	-1722.07604322
Thermal and entropic correction, BS1 (a.u.)	0.335725
Electronic Energy, BS2 (a.u.)	-3165.9283957

C	-1.979184	-0.720057	-0.609541
C	-0.815398	0.037547	0.014317

H	-1.774953	-0.877979	-1.676786
H	-1.039851	0.257230	1.066145
H	-0.684617	0.993430	-0.493317
C	-0.955392	-2.783803	0.018714
C	-1.169632	-4.147822	0.129490
C	-0.059881	-5.003542	0.151713
C	1.231460	-4.503181	0.041772
C	1.469537	-3.116836	-0.066297
C	0.348559	-2.205748	-0.064151
H	-2.178155	-4.538772	0.186894
H	2.085894	-5.173122	0.045352
C	0.461246	-0.764240	-0.051966
N	1.619381	-0.149934	-0.047012
O	-2.093340	-2.019348	0.017620
O	-0.317478	-6.342137	0.270095
H	0.519536	-6.831960	0.272889
C	-3.298191	-0.009914	-0.442160
C	-3.904116	0.056576	0.822849
C	-3.905454	0.621784	-1.526347
C	-5.099331	0.741212	0.991322
H	-3.455693	-0.432771	1.681344
C	-5.105868	1.326282	-1.363337
H	-3.446790	0.571444	-2.509394
C	-5.706597	1.388241	-0.108197
H	-5.564294	1.812866	-2.215866
O	-5.684251	0.784528	2.234062
H	-6.510006	1.289887	2.157441
O	-6.878855	2.033521	0.193643
C	-7.570122	2.699826	-0.867410
H	-7.863810	1.993137	-1.651793
H	-8.462024	3.136004	-0.415977
H	-6.953380	3.494444	-1.302385
O	2.729984	-2.740471	-0.162425
N	1.640766	1.234207	0.015851
C	2.879398	1.682145	0.051089
O	3.942535	0.918062	0.040663
C	3.084766	3.153776	0.106826
C	4.389028	3.671035	0.155603
C	1.997876	4.046076	0.106644
C	4.603091	5.048985	0.202721
H	5.231504	2.988422	0.156092

C	2.214853	5.421314	0.153646
H	0.987711	3.654757	0.068507
C	3.517938	5.928650	0.201560
H	5.617633	5.434907	0.239833
H	1.366546	6.099591	0.152143
H	3.684628	7.001294	0.237206
Cu	3.394747	-0.957917	-0.078943
O	5.329952	-1.687768	-0.151127
H	5.864473	-1.116048	-0.724516
H	5.307060	-2.548013	-0.599760

Table S2.3. $[(H_2O)_{14}\cdot H]^+_{aq}$

Electronic Energy, BS1 (a.u.)	-1070.74424028
Thermal and entropic correction, BS1 (a.u.)	0.303167
Electronic Energy, BS2 (a.u.)	-1071.25351115

O	-3.693781	-0.161886	0.716538
H	-4.628152	-0.005772	0.910816
H	-3.353542	0.670353	0.292993
O	-0.397246	2.694948	0.910859
H	-1.156570	2.421508	0.338725
H	0.371205	2.772088	0.303536
O	2.306254	-0.440089	1.826987
H	2.843844	0.089266	1.193427
H	2.173912	-1.299727	1.367547
O	3.716574	0.874471	-0.125101
H	4.669190	1.010915	-0.030796
H	3.595566	0.146887	-0.784477
O	-1.780768	0.376327	-2.591079
H	-2.226209	-0.440604	-2.275334
H	-0.864308	0.280993	-2.238834
O	-0.522488	-1.925857	0.090930
H	-0.169397	-1.067483	-0.253648
H	-1.313519	-2.098188	-0.471031
O	-2.534707	1.883597	-0.549707

H	-3.111101	2.604302	-0.841160
H	-2.274344	1.382585	-1.379919
O	-1.665551	-0.995306	2.336885
H	-1.247140	-1.477611	1.577756
H	-2.473903	-0.619941	1.916243
O	2.906245	-1.150760	-1.676251
H	2.687503	-1.870320	-1.032768
H	3.236431	-1.576053	-2.478802
O	2.002246	-2.722779	0.286605
H	1.022106	-2.666118	0.162345
H	2.201206	-3.624498	0.572197
O	-2.864171	-1.799297	-1.268518
H	-3.304625	-1.283537	-0.547314
H	-3.495642	-2.476693	-1.545592
O	0.560910	0.162015	-1.225839
H	0.876022	1.044454	-0.936612
H	1.365740	-0.296449	-1.560593
O	1.789818	2.633956	-0.718306
H	2.595781	2.099283	-0.509471
H	1.848660	2.849723	-1.659046
O	0.153278	0.762771	2.433968
H	-0.627191	0.077893	2.416555
H	1.013919	0.274230	2.130752
H	-0.068345	1.541629	1.778743

Section S3. [Cu(L^{3}H$_2$-κS)(AcO)]$_{aq}$ ⇄ [Cu(L^{3}H$_2$-κN)(AcO)]$_{aq}$

This is Equation (4) of the main text.
In the next tables, for each molecule BS1 refers to the basis set 6-31G(d,p), applied to the main group elements, and the SDD plus f-functions, employed for the metal; BS2 refers to the basis set def2-TZVP for main group and def2-QZVP for d-block elements (see section 3.3 of the main text).

Table S3.1. [Cu(L^{3}H$_2$-κS)(AcO)]$_{aq}$

Electronic Energy, BS1 (a.u.)	-2022.01285334
Thermal and entropic correction, BS1 (a.u.)	0.305947

C	-1.979018	0.475960	0.476524
C	-1.152370	-0.461962	-0.396095
H	-1.582620	0.449551	1.499766
H	-1.570352	-0.492007	-1.410779
H	-1.229305	-1.466234	0.028172
C	-0.563611	2.278959	-0.164439
C	-0.430667	3.654057	-0.179558
C	0.849465	4.207714	-0.341443
C	1.971631	3.399979	-0.466716
C	1.859854	1.992871	-0.448491
C	0.550528	1.389995	-0.313590
H	-1.299858	4.287792	-0.054149
H	2.959837	3.836454	-0.573347
C	0.290617	-0.016370	-0.455288
N	1.253429	-0.892892	-0.669793
O	-1.845328	1.824212	-0.020051
O	0.924795	5.570169	-0.344356
H	1.850373	5.843113	-0.440340
C	-3.444060	0.123810	0.486235
C	-4.230495	0.356165	-0.653401
C	-4.019438	-0.470531	1.608178

C	-5.571764	0.000744	-0.660508
H	-3.807448	0.823017	-1.536932
C	-5.370326	-0.842607	1.605909
H	-3.418836	-0.649499	2.494974
C	-6.150247	-0.608115	0.475889
H	-5.802072	-1.303455	2.486189
O	-6.329891	0.243642	-1.780134
H	-7.230307	-0.075245	-1.604652
O	-7.479797	-0.912206	0.336158
C	-8.145152	-1.538287	1.438099
H	-8.130891	-0.894170	2.324576
H	-9.175540	-1.693554	1.116188
H	-7.685780	-2.503767	1.678439
O	2.966845	1.304051	-0.571820
N	0.871164	-2.200696	-0.940161
H	-0.012840	-2.357112	-1.415948
C	1.749826	-3.215224	-0.851137
C	5.313443	-0.182707	1.401198
O	4.423541	-0.148035	2.275210
O	5.089825	-0.373068	0.136165
C	6.775274	-0.007697	1.768664
H	7.203392	0.823092	1.199478
H	7.330103	-0.911055	1.495582
H	6.887883	0.181028	2.837239
Cu	3.195437	-0.602431	-0.315508
N	1.321400	-4.427815	-1.205382
H	0.359465	-4.592366	-1.478360
H	1.946496	-5.219027	-1.149318
S	3.362364	-2.953554	-0.304328

Table S3.2. [Cu(L^3H_2-κN)(AcO)]$_{aq}$

Electronic Energy, BS1 (a.u.) -2022.00121328

Thermal and entropic correction, BS1 (a.u.) 0.306182

C	-2.047565	0.552053	0.534764

C	-1.119183	-0.401116	-0.208109
H	-1.710076	0.638938	1.575279
H	-1.485523	-0.560636	-1.230750
H	-1.139258	-1.365283	0.307949
C	-0.699983	2.390592	-0.149658
C	-0.643590	3.768612	-0.228512
C	0.612916	4.386379	-0.331741
C	1.784264	3.639934	-0.336409
C	1.752310	2.232417	-0.255302
C	0.469591	1.563033	-0.178887
H	-1.553072	4.355788	-0.199358
H	2.750824	4.130117	-0.398409
C	0.288137	0.140569	-0.262015
N	1.308156	-0.672656	-0.433182
O	-1.959559	1.861581	-0.072061
O	0.617969	5.747957	-0.405605
H	1.533510	6.065194	-0.448577
C	-3.488910	0.115938	0.499900
C	-4.195152	0.120301	-0.713877
C	-4.121927	-0.325110	1.660841
C	-5.513573	-0.309623	-0.756637
H	-3.727387	0.464316	-1.630832
C	-5.450612	-0.768518	1.625899
H	-3.583491	-0.328028	2.603782
C	-6.150495	-0.760791	0.421684
H	-5.928597	-1.108759	2.536679
O	-6.191304	-0.294423	-1.951465
H	-7.086014	-0.636669	-1.791536
O	-7.450127	-1.158416	0.243154
C	-8.169957	-1.632054	1.386034
H	-8.250914	-0.852180	2.151636
H	-9.165170	-1.894166	1.025065
H	-7.689638	-2.518834	1.814679
O	2.912169	1.613988	-0.254911
N	1.059113	-2.021226	-0.653943
H	0.181482	-2.300342	-1.082811
C	2.063348	-2.913772	-0.633881
C	5.626453	-0.568180	1.152821
O	4.918651	-1.085511	2.040150
O	5.162965	-0.077761	0.040032
C	7.131446	-0.470667	1.309846

H	7.443661	0.574866	1.226487
H	7.620094	-1.022575	0.500559
H	7.448785	-0.874213	2.272336
Cu	3.237527	-0.275554	-0.216733
N	3.254910	-2.373341	-0.065466
H	3.235663	-2.404164	0.960260
H	4.076556	-2.892129	-0.368903
S	1.933363	-4.464307	-1.237817

Section S4. Estimation of the rotational barrier

The rotational barrier ($\Delta E^{‡}$ in Figure S1) was estimated through scan analysis of the dihedral O–Cu-O1-O2 (Figure S1). The ΔG of the rotation was estimated by the energy difference between the species **I** and **II**, each one corrected with the thermal and entropic contribution obtained from the frequency calculation (for **I** see $[Cu(L^1H_2)(AcO)]_{aq}$ Table S1.1, for **II** see the Cartesian coordinates scheme below in this subsection).

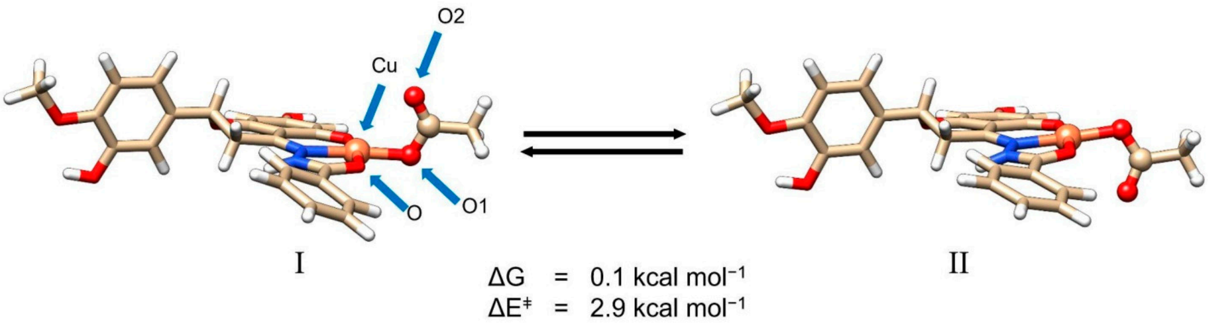

Figure S1. Scheme of reaction of the rotation of the monodentate acetate ligand around the bond Cu–O1. The values of ΔG and of the energy barrier $\Delta E^{‡}$ for the flipping of the acetato ligand are also shown.

Table S4.1. **II**

Electronic Energy, BS1 (a.u.) -1874.751477
Thermal and entropic correction, BS1 (a.u.) 0.368563

C	-2.410011	-0.887188	-0.547357
C	-1.310799	0.019887	-0.007211
H	-2.212343	-1.092758	-1.607046
H	-1.535227	0.309247	1.027676
H	-1.297052	0.931119	-0.612785
C	-1.171022	-2.802808	0.116048

C	-1.236000	-4.172820	0.276296
C	-0.041302	-4.910990	0.248904
C	1.184217	-4.291487	0.041576
C	1.275421	-2.892531	-0.120008
C	0.064689	-2.100698	-0.059285
H	-2.192118	-4.664372	0.406966
H	2.098393	-4.875313	-0.000530
C	0.033542	-0.664884	-0.051300
N	1.142891	0.038902	-0.035055
O	-2.371419	-2.146269	0.164318
O	-0.157570	-6.259676	0.414402
H	0.720180	-6.668467	0.361734
C	-3.789813	-0.304367	-0.390765
C	-4.345422	-0.144170	0.889027
C	-4.514661	0.102349	-1.509755
C	-5.608090	0.411461	1.037548
H	-3.805083	-0.460044	1.775698
C	-5.786060	0.674121	-1.368831
H	-4.093770	-0.023127	-2.502843
C	-6.337016	0.828923	-0.099052
H	-6.336445	0.985647	-2.248400
O	-6.141711	0.552867	2.295415
H	-7.023873	0.948293	2.201301
O	-7.567040	1.362582	0.185630
C	-8.386893	1.780479	-0.910721
H	-8.616103	0.938757	-1.573895
H	-9.308847	2.158630	-0.467572
H	-7.901589	2.578095	-1.484389
O	2.473667	-2.396023	-0.327724
N	1.076170	1.417754	0.061939
H	0.255249	1.861090	0.459622
C	2.229579	2.098320	-0.093733
O	3.296258	1.469580	-0.352924
C	2.212961	3.567891	0.041111
C	3.419197	4.214663	0.354885
C	1.044325	4.322606	-0.157636
C	3.450741	5.600621	0.486688
H	4.317509	3.625162	0.502258
C	1.085369	5.709197	-0.028296
H	0.113993	3.840286	-0.440742
C	2.285113	6.349043	0.296520

H	4.382856	6.097199	0.737150
H	0.183268	6.290295	-0.191019
H	2.312537	7.429933	0.395702
C	5.681588	-0.935617	0.196136
O	5.341304	-0.673471	1.367289
O	4.861177	-0.973953	-0.810430
C	7.126412	-1.244247	-0.148031
H	7.478829	-0.551705	-0.918585
H	7.195110	-2.255418	-0.561669
H	7.762026	-1.166879	0.735235
Cu	3.005357	-0.547721	-0.366428

Section S5. Docking calculations

Section S5.1. Docking ligand structures

In this section, the MOL2 files of each ligand structure implemented for the docking calculations is presented. The scoring function GoldScore was implemented with the GoldScore and GOLD parameter files used by Sciortino et al. [1]. DFT-computed energies come along with the coordinates for those structures that have not been described in the previous section.
All the docking ligand structures are summarized in Table 4 of the manuscript.

Table S5.1. (*R*)-$L^1H_3^{am}$ (HHSB; $C_{23}N_2H_{20}O_6$)

Electronic Energy, BS1 (a.u.)	-1449.40950886
Thermal and entropic correction, BS1 (a.u.)	0.336636

```
@<TRIPOS>MOLECULE
C23N2H20O6
51 54 1 0 0
r-HHSB
NO_CHARGES


@<TRIPOS>ATOM
      1 C1          1.5462    0.5444   -0.5900 C.3       1 UNK    0.0000
      2 C2          0.2270    0.0790    0.0314 C.3       1 UNK    0.0000
      3 H1          1.3863    0.7331   -1.6590 H         1 UNK    0.0000
      4 H2          0.3849   -0.1790    1.0862 H         1 UNK    0.0000
      5 H3         -0.0872   -0.8308   -0.4889 H         1 UNK    0.0000
      6 C3          1.0442    2.8044   -0.0038 C.ar      1 UNK    0.0000
      7 C4          1.5454    4.0977    0.0739 C.ar      1 UNK    0.0000
      8 C5          0.6463    5.1702    0.0685 C.ar      1 UNK    0.0000
      9 C6         -0.7323    4.9603   -0.0235 C.ar      1 UNK    0.0000
     10 C7         -1.2254    3.6578   -0.0960 C.ar      1 UNK    0.0000
     11 C8         -0.3442    2.5373   -0.0846 C.ar      1 UNK    0.0000
     12 H4          2.6138    4.2688    0.1256 H         1 UNK    0.0000
     13 H5         -1.4257    5.7950   -0.0312 H         1 UNK    0.0000
     14 C9         -0.8174    1.1613   -0.0658 C.2       1 UNK    0.0000
     15 N1         -2.1018    0.9686   -0.0979 N.2       1 UNK    0.0000
     16 O1          1.9670    1.7921    0.0188 O.3       1 UNK    0.0000
```

```
     17 O2          1.1818    6.4253    0.1430 O.3       1 UNK    0.0000
     18 H6          0.4703    7.0831    0.1055 H         1 UNK    0.0000
     19 C10         2.6585   -0.4551   -0.4079 C.ar      1 UNK    0.0000
     20 C11         3.1971   -0.6814    0.8691 C.ar      1 UNK    0.0000
     21 C12         3.1291   -1.1945   -1.4919 C.ar      1 UNK    0.0000
     22 C13         4.1916   -1.6320    1.0494 C.ar      1 UNK    0.0000
     23 H7          2.8517   -0.1154    1.7282 H         1 UNK    0.0000
     24 C14         4.1262   -2.1636   -1.3175 C.ar      1 UNK    0.0000
     25 H8          2.7200   -1.0234   -2.4831 H         1 UNK    0.0000
     26 C15         4.6606   -2.3849   -0.0505 C.ar      1 UNK    0.0000
     27 H9          4.4800   -2.7311   -2.1699 H         1 UNK    0.0000
     28 O3          4.7131   -1.8382    2.3037 O.3       1 UNK    0.0000
     29 H10         5.3854   -2.5358    2.2333 H         1 UNK    0.0000
     30 O4          5.6381   -3.2930    0.2652 O.3       1 UNK    0.0000
     31 C16         6.1592   -4.1114   -0.7870 C.3       1 UNK    0.0000
     32 H11         6.6361   -3.5008   -1.5620 H         1 UNK    0.0000
     33 H12         6.9043   -4.7580   -0.3219 H         1 UNK    0.0000
     34 H13         5.3702   -4.7245   -1.2368 H         1 UNK    0.0000
     35 N2         -2.6164   -0.2930   -0.0253 N.am      1 UNK    0.0000
     36 H14        -2.0160   -1.0798    0.1971 H         1 UNK    0.0000
     37 C17        -3.9751   -0.4560   -0.0794 C.2       1 UNK    0.0000
     38 O5         -4.7467    0.5071   -0.1807 O.2       1 UNK    0.0000
     39 C18        -4.4630   -1.8651    0.0045 C.ar      1 UNK    0.0000
     40 C19        -5.7750   -2.0671    0.4596 C.ar      1 UNK    0.0000
     41 C20        -3.6862   -2.9722   -0.3748 C.ar      1 UNK    0.0000
     42 C21        -6.2955   -3.3556    0.5573 C.ar      1 UNK    0.0000
     43 H15        -6.3736   -1.2068    0.7396 H         1 UNK    0.0000
     44 C22        -4.2134   -4.2604   -0.2818 C.ar      1 UNK    0.0000
     45 H16        -2.6833   -2.8397   -0.7695 H         1 UNK    0.0000
     46 C23        -5.5149   -4.4553    0.1880 C.ar      1 UNK    0.0000
     47 H17        -7.3083   -3.5027    0.9197 H         1 UNK    0.0000
     48 H18        -3.6098   -5.1105   -0.5843 H         1 UNK    0.0000
     49 H19        -5.9212   -5.4597    0.2605 H         1 UNK    0.0000
     50 O6         -2.5703    3.4977   -0.1677 O.3       1 UNK    0.0000
     51 H20        -2.7505    2.5139   -0.1680 H         1 UNK    0.0000
@<TRIPOS>BOND
     1    1    2 1
     2    1    3 1
     3    1   16 1
     4    1   19 1
     5    2    4 1
     6    2    5 1
     7    2   14 1
     8    6    7 ar
     9    6   11 ar
    10    6   16 1
    11    7    8 ar
    12    7   12 1
    13    8    9 ar
```

```
    14    8   17 1
    15    9   10 ar
    16    9   13 1
    17   10   11 ar
    18   10   50 1
    19   11   14 1
    20   14   15 2
    21   15   35 1
    22   17   18 1
    23   19   20 ar
    24   19   21 ar
    25   20   22 ar
    26   20   23 1
    27   21   24 ar
    28   21   25 1
    29   22   26 ar
    30   22   28 1
    31   24   26 ar
    32   24   27 1
    33   26   30 1
    34   28   29 1
    35   30   31 1
    36   31   32 1
    37   31   33 1
    38   31   34 1
    39   35   36 1
    40   35   37 am
    41   37   38 2
    42   37   39 1
    43   39   40 ar
    44   39   41 ar
    45   40   42 ar
    46   40   43 1
    47   41   44 ar
    48   41   45 1
    49   42   46 ar
    50   42   47 1
    51   44   46 ar
    52   44   48 1
    53   46   49 1
    54   50   51 1
@<TRIPOS>SUBSTRUCTURE
     1 UNK      1 RESIDUE           4 A      UNK      0 ROOT
```

Table S5.2. (*S*)-$L^{1}H_{3}^{am}$ (HHSB; $C_{23}N_{2}H_{20}O_{6}$)

Electronic Energy, BS1 (a.u.) -1449.40950886

Thermal and entropic correction, BS1 (a.u.) 0.336636

```
@<TRIPOS>MOLECULE
C23N2H20O6
51 54 1 0 0
s-HHSB
NO_CHARGES


@<TRIPOS>ATOM
      1 C1          1.5462    0.5444    0.5900 C.3       1 UNK    0.0000
      2 C2          0.2270    0.0790   -0.0314 C.3       1 UNK    0.0000
      3 H1          1.3863    0.7331    1.6590 H         1 UNK    0.0000
      4 H2          0.3849   -0.1790   -1.0862 H         1 UNK    0.0000
      5 H3         -0.0872   -0.8308    0.4889 H         1 UNK    0.0000
      6 C3          1.0442    2.8044    0.0038 C.ar      1 UNK    0.0000
      7 C4          1.5454    4.0977   -0.0739 C.ar      1 UNK    0.0000
      8 C5          0.6463    5.1702   -0.0685 C.ar      1 UNK    0.0000
      9 C6         -0.7323    4.9603    0.0235 C.ar      1 UNK    0.0000
     10 C7         -1.2254    3.6578    0.0960 C.ar      1 UNK    0.0000
     11 C8         -0.3442    2.5373    0.0846 C.ar      1 UNK    0.0000
     12 H4          2.6138    4.2688   -0.1256 H         1 UNK    0.0000
     13 H5         -1.4257    5.7950    0.0312 H         1 UNK    0.0000
     14 C9         -0.8174    1.1613    0.0658 C.2       1 UNK    0.0000
     15 N1         -2.1018    0.9686    0.0979 N.2       1 UNK    0.0000
     16 O1          1.9670    1.7921   -0.0188 O.3       1 UNK    0.0000
     17 O2          1.1818    6.4253   -0.1430 O.3       1 UNK    0.0000
     18 H6          0.4703    7.0831   -0.1055 H         1 UNK    0.0000
     19 C10         2.6585   -0.4551    0.4079 C.ar      1 UNK    0.0000
     20 C11         3.1971   -0.6814   -0.8691 C.ar      1 UNK    0.0000
     21 C12         3.1291   -1.1945    1.4919 C.ar      1 UNK    0.0000
     22 C13         4.1916   -1.6320   -1.0494 C.ar      1 UNK    0.0000
     23 H7          2.8517   -0.1154   -1.7282 H         1 UNK    0.0000
     24 C14         4.1262   -2.1636    1.3175 C.ar      1 UNK    0.0000
     25 H8          2.7200   -1.0234    2.4831 H         1 UNK    0.0000
     26 C15         4.6606   -2.3849    0.0505 C.ar      1 UNK    0.0000
     27 H9          4.4800   -2.7311    2.1699 H         1 UNK    0.0000
     28 O3          4.7131   -1.8382   -2.3037 O.3       1 UNK    0.0000
     29 H10         5.3854   -2.5358   -2.2333 H         1 UNK    0.0000
     30 O4          5.6381   -3.2930   -0.2652 O.3       1 UNK    0.0000
     31 C16         6.1592   -4.1114    0.7870 C.3       1 UNK    0.0000
     32 H11         6.6361   -3.5008    1.5620 H         1 UNK    0.0000
     33 H12         6.9043   -4.7580    0.3219 H         1 UNK    0.0000
     34 H13         5.3702   -4.7245    1.2368 H         1 UNK    0.0000
```

```
      35 N2          -2.6164   -0.2930    0.0253 N.am       1 UNK    0.0000
      36 H14         -2.0160   -1.0798   -0.1971 H          1 UNK    0.0000
      37 C17         -3.9751   -0.4560    0.0794 C.2        1 UNK    0.0000
      38 O5          -4.7467    0.5071    0.1807 O.2        1 UNK    0.0000
      39 C18         -4.4630   -1.8651   -0.0045 C.ar       1 UNK    0.0000
      40 C19         -5.7750   -2.0671   -0.4596 C.ar       1 UNK    0.0000
      41 C20         -3.6862   -2.9722    0.3748 C.ar       1 UNK    0.0000
      42 C21         -6.2955   -3.3556   -0.5573 C.ar       1 UNK    0.0000
      43 H15         -6.3736   -1.2068   -0.7396 H          1 UNK    0.0000
      44 C22         -4.2134   -4.2604    0.2818 C.ar       1 UNK    0.0000
      45 H16         -2.6833   -2.8397    0.7695 H          1 UNK    0.0000
      46 C23         -5.5149   -4.4553   -0.1880 C.ar       1 UNK    0.0000
      47 H17         -7.3083   -3.5027   -0.9197 H          1 UNK    0.0000
      48 H18         -3.6098   -5.1105    0.5843 H          1 UNK    0.0000
      49 H19         -5.9212   -5.4597   -0.2605 H          1 UNK    0.0000
      50 O6          -2.5703    3.4977    0.1677 O.3        1 UNK    0.0000
      51 H20         -2.7505    2.5139    0.1680 H          1 UNK    0.0000
@<TRIPOS>BOND
     1    1    2 1
     2    1    3 1
     3    1   16 1
     4    1   19 1
     5    2    4 1
     6    2    5 1
     7    2   14 1
     8    6    7 ar
     9    6   11 ar
    10    6   16 1
    11    7    8 ar
    12    7   12 1
    13    8    9 ar
    14    8   17 1
    15    9   10 ar
    16    9   13 1
    17   10   11 ar
    18   10   50 1
    19   11   14 1
    20   14   15 2
    21   15   35 1
    22   17   18 1
    23   19   20 ar
    24   19   21 ar
    25   20   22 ar
    26   20   23 1
    27   21   24 ar
    28   21   25 1
    29   22   26 ar
    30   22   28 1
    31   24   26 ar
```

```
    32   24   27 1
    33   26   30 1
    34   28   29 1
    35   30   31 1
    36   31   32 1
    37   31   33 1
    38   31   34 1
    39   35   36 1
    40   35   37 am
    41   37   38 2
    42   37   39 1
    43   39   40 ar
    44   39   41 ar
    45   40   42 ar
    46   40   43 1
    47   41   44 ar
    48   41   45 1
    49   42   46 ar
    50   42   47 1
    51   44   46 ar
    52   44   48 1
    53   46   49 1
    54   50   51 1
@<TRIPOS>SUBSTRUCTURE
     1 UNK     1 RESIDUE           4 A     UNK     0 ROOT
```

Table S5.3. [Cu((*R*)-L[1]H_2^{am})(AcO)] (CuHHSB; $CuC_{25}N_2H_{22}O_8$)

Electronic Energy, BS1 (a.u.)	-1874.7512616
Thermal and entropic correction, BS1 (a.u.)	0.368166
Electronic Energy, BS2 (a.u.)	-3318.66519038

```
@<TRIPOS>MOLECULE
CuC25N2H22O8
58 63 1 0 0
Cu_r-HHSB
NO_CHARGES


@<TRIPOS>ATOM
      1 C1         -2.3494   -0.9062   -0.5479 C.3       1 UNK    0.0000
```

2	C2	-1.2889	0.0088	0.0534	C.3	1	UNK	0.0000
3	H1	-2.0826	-1.1238	-1.5897	H	1	UNK	0.0000
4	H2	-1.5930	0.3299	1.0582	H	1	UNK	0.0000
5	H3	-1.2253	0.9009	-0.5769	H	1	UNK	0.0000
6	C3	-1.1655	-2.8128	0.2388	C.ar	1	UNK	0.0000
7	C4	-1.2478	-4.1819	0.3963	C.ar	1	UNK	0.0000
8	C5	-0.0581	-4.9231	0.4874	C.ar	1	UNK	0.0000
9	C6	1.1828	-4.3049	0.4091	C.ar	1	UNK	0.0000
10	C7	1.2919	-2.9060	0.2549	C.ar	1	UNK	0.0000
11	C8	0.0826	-2.1128	0.1762	C.ar	1	UNK	0.0000
12	H4	-2.2131	-4.6712	0.4350	H	1	UNK	0.0000
13	H5	2.0964	-4.8882	0.4669	H	1	UNK	0.0000
14	C9	0.0528	-0.6773	0.1423	C.2	1	UNK	0.0000
15	N1	1.1574	0.0291	0.2319	N.pl3	1	UNK	0.0000
16	O1	-2.3654	-2.1563	0.1779	O.3	1	UNK	0.0000
17	O2	-0.1958	-6.2715	0.6385	O.3	1	UNK	0.0000
18	H6	0.6797	-6.6875	0.6651	H	1	UNK	0.0000
19	C10	-3.7335	-0.3144	-0.4856	C.ar	1	UNK	0.0000
20	C11	-4.3712	-0.1543	0.7552	C.ar	1	UNK	0.0000
21	C12	-4.3769	0.1075	-1.6478	C.ar	1	UNK	0.0000
22	C13	-5.6338	0.4165	0.8233	C.ar	1	UNK	0.0000
23	H7	-3.8945	-0.4810	1.6739	H	1	UNK	0.0000
24	C14	-5.6478	0.6949	-1.5877	C.ar	1	UNK	0.0000
25	H8	-3.8921	-0.0176	-2.6114	H	1	UNK	0.0000
26	C15	-6.2801	0.8502	-0.3564	C.ar	1	UNK	0.0000
27	H9	-6.1353	1.0182	-2.4995	H	1	UNK	0.0000
28	O3	-6.2478	0.5576	2.0440	O.3	1	UNK	0.0000
29	H10	-7.1134	0.9722	1.8953	H	1	UNK	0.0000
30	O4	-7.5196	1.3985	-0.1507	O.3	1	UNK	0.0000
31	C16	-8.2435	1.8657	-1.2938	C.3	1	UNK	0.0000
32	H11	-8.4462	1.0471	-1.9934	H	1	UNK	0.0000
33	H12	-9.1852	2.2606	-0.9106	H	1	UNK	0.0000
34	H13	-7.6942	2.6614	-1.8094	H	1	UNK	0.0000
35	O5	2.5070	-2.4118	0.1888	O.pl3	1	UNK	0.0000
36	N2	1.0864	1.4100	0.2754	N.pl3	1	UNK	0.0000
37	H14	0.2227	1.8731	0.5355	H	1	UNK	0.0000
38	C17	2.2526	2.0844	0.2469	C.2	1	UNK	0.0000
39	O6	3.3419	1.4463	0.1674	O.pl3	1	UNK	0.0000
40	C18	2.2216	3.5581	0.3109	C.ar	1	UNK	0.0000
41	C19	3.3622	4.2217	0.7909	C.ar	1	UNK	0.0000
42	C20	1.1048	4.2989	-0.1118	C.ar	1	UNK	0.0000
43	C21	3.3764	5.6120	0.8673	C.ar	1	UNK	0.0000
44	H15	4.2217	3.6420	1.1093	H	1	UNK	0.0000
45	C22	1.1293	5.6900	-0.0365	C.ar	1	UNK	0.0000
46	H16	0.2332	3.8024	-0.5265	H	1	UNK	0.0000
47	C23	2.2606	6.3473	0.4558	C.ar	1	UNK	0.0000
48	H17	4.2560	6.1219	1.2474	H	1	UNK	0.0000
49	H18	0.2690	6.2608	-0.3711	H	1	UNK	0.0000
50	H19	2.2748	7.4315	0.5126	H	1	UNK	0.0000

```
     51 C24          5.4999   -0.9170   -1.0459 C.2       1 UNK    0.0000
     52 O7           4.8770   -0.6728   -2.0987 O.2       1 UNK    0.0000
     53 O8           4.9510   -0.9713    0.1312 O.pl3     1 UNK    0.0000
     54 C25          6.9949   -1.1738   -1.0626 C.3       1 UNK    0.0000
     55 H20          7.2087   -2.1437   -0.6034 H         1 UNK    0.0000
     56 H21          7.5024   -0.4103   -0.4643 H         1 UNK    0.0000
     57 H22          7.3818   -1.1550   -2.0824 H         1 UNK    0.0000
     58 Cu1          3.0393   -0.5654    0.1622 M.Cu      1 UNK    0.0000
@<TRIPOS>BOND
     1    1    2 1
     2    1    3 1
     3    1   16 1
     4    1   19 1
     5    2    4 1
     6    2    5 1
     7    2   14 1
     8    6    7 ar
     9    6   11 ar
    10    6   16 1
    11    7    8 ar
    12    7   12 1
    13    8    9 ar
    14    8   17 1
    15    9   10 ar
    16    9   13 1
    17   10   11 ar
    18   10   35 1
    19   11   14 1
    20   14   15 2
    21   15   36 2
    22   17   18 1
    23   19   20 ar
    24   19   21 ar
    25   20   22 ar
    26   20   23 1
    27   21   24 ar
    28   21   25 1
    29   22   26 ar
    30   22   28 1
    31   24   26 ar
    32   24   27 1
    33   26   30 1
    34   28   29 1
    35   30   31 1
    36   31   32 1
    37   31   33 1
    38   31   34 1
    39   36   37 1
    40   36   38 2
```

```
    41   38   39 1
    42   38   40 1
    43   40   41 ar
    44   40   42 ar
    45   41   43 ar
    46   41   44 1
    47   42   45 ar
    48   42   46 1
    49   43   47 ar
    50   43   48 1
    51   45   47 ar
    52   45   49 1
    53   47   50 1
    54   51   52 2
    55   51   53 1
    56   51   54 1
    57   54   55 1
    58   54   56 1
    59   54   57 1
    60   15   58 1
    61   35   58 1
    62   39   58 1
    63   53   58 1
@<TRIPOS>SUBSTRUCTURE
     1 UNK     1 RESIDUE           4 A     UNK     0 ROOT
```

Table S5.4. [Cu((*S*)-$L^1H_2^{am}$)(AcO)] (CuHHSB; $CuC_{25}N_2H_{22}O_8$)

Electronic Energy, BS1 (a.u.)	-1874.7512616
Thermal and entropic correction, BS1 (a.u.)	0.368166
Electronic Energy, BS2 (a.u.)	-3318.66519038

```
@<TRIPOS>MOLECULE
CuC25N2H22O8
58 63 1 0 0
Cu_s-HHSB
NO_CHARGES


@<TRIPOS>ATOM
      1 C1         -2.3494   -0.9062    0.5479 C.3       1 UNK    0.0000
```

2	C2	-1.2889	0.0088	-0.0534	C.3	1	UNK	0.0000
3	H1	-2.0826	-1.1238	1.5897	H	1	UNK	0.0000
4	H2	-1.5930	0.3299	-1.0582	H	1	UNK	0.0000
5	H3	-1.2253	0.9009	0.5769	H	1	UNK	0.0000
6	C3	-1.1655	-2.8128	-0.2388	C.ar	1	UNK	0.0000
7	C4	-1.2478	-4.1819	-0.3963	C.ar	1	UNK	0.0000
8	C5	-0.0581	-4.9231	-0.4874	C.ar	1	UNK	0.0000
9	C6	1.1828	-4.3049	-0.4091	C.ar	1	UNK	0.0000
10	C7	1.2919	-2.9060	-0.2549	C.ar	1	UNK	0.0000
11	C8	0.0826	-2.1128	-0.1762	C.ar	1	UNK	0.0000
12	H4	-2.2131	-4.6712	-0.4350	H	1	UNK	0.0000
13	H5	2.0964	-4.8882	-0.4669	H	1	UNK	0.0000
14	C9	0.0528	-0.6773	-0.1423	C.2	1	UNK	0.0000
15	N1	1.1574	0.0291	-0.2319	N.pl3	1	UNK	0.0000
16	O1	-2.3654	-2.1563	-0.1779	O.3	1	UNK	0.0000
17	O2	-0.1958	-6.2715	-0.6385	O.3	1	UNK	0.0000
18	H6	0.6797	-6.6875	-0.6651	H	1	UNK	0.0000
19	C10	-3.7335	-0.3144	0.4856	C.ar	1	UNK	0.0000
20	C11	-4.3712	-0.1543	-0.7552	C.ar	1	UNK	0.0000
21	C12	-4.3769	0.1075	1.6478	C.ar	1	UNK	0.0000
22	C13	-5.6338	0.4165	-0.8233	C.ar	1	UNK	0.0000
23	H7	-3.8945	-0.4810	-1.6739	H	1	UNK	0.0000
24	C14	-5.6478	0.6949	1.5877	C.ar	1	UNK	0.0000
25	H8	-3.8921	-0.0176	2.6114	H	1	UNK	0.0000
26	C15	-6.2801	0.8502	0.3564	C.ar	1	UNK	0.0000
27	H9	-6.1353	1.0182	2.4995	H	1	UNK	0.0000
28	O3	-6.2478	0.5576	-2.0440	O.3	1	UNK	0.0000
29	H10	-7.1134	0.9722	-1.8953	H	1	UNK	0.0000
30	O4	-7.5196	1.3985	0.1507	O.3	1	UNK	0.0000
31	C16	-8.2435	1.8657	1.2938	C.3	1	UNK	0.0000
32	H11	-8.4462	1.0471	1.9934	H	1	UNK	0.0000
33	H12	-9.1852	2.2606	0.9106	H	1	UNK	0.0000
34	H13	-7.6942	2.6614	1.8094	H	1	UNK	0.0000
35	O5	2.5070	-2.4118	-0.1888	O.2cor	1	UNK	0.0000
36	N2	1.0864	1.4100	-0.2754	N.am	1	UNK	0.0000
37	H14	0.2227	1.8731	-0.5355	H	1	UNK	0.0000
38	C17	2.2526	2.0844	-0.2469	C.2	1	UNK	0.0000
39	O6	3.3419	1.4463	-0.1674	O.2cor	1	UNK	0.0000
40	C18	2.2216	3.5581	-0.3109	C.ar	1	UNK	0.0000
41	C19	3.3622	4.2217	-0.7909	C.ar	1	UNK	0.0000
42	C20	1.1048	4.2989	0.1118	C.ar	1	UNK	0.0000
43	C21	3.3764	5.6120	-0.8673	C.ar	1	UNK	0.0000
44	H15	4.2217	3.6420	-1.1093	H	1	UNK	0.0000
45	C22	1.1293	5.6900	0.0365	C.ar	1	UNK	0.0000
46	H16	0.2332	3.8024	0.5265	H	1	UNK	0.0000
47	C23	2.2606	6.3473	-0.4558	C.ar	1	UNK	0.0000
48	H17	4.2560	6.1219	-1.2474	H	1	UNK	0.0000
49	H18	0.2690	6.2608	0.3711	H	1	UNK	0.0000
50	H19	2.2748	7.4315	-0.5126	H	1	UNK	0.0000

```
     51 C24          5.4999   -0.9170    1.0459 C.2        1 UNK    0.0000
     52 O7           4.8770   -0.6728    2.0987 O.2        1 UNK    0.0000
     53 O8           4.9510   -0.9713   -0.1312 O.2cor     1 UNK    0.0000
     54 C25          6.9949   -1.1738    1.0626 C.3        1 UNK    0.0000
     55 H20          7.2087   -2.1437    0.6034 H          1 UNK    0.0000
     56 H21          7.5024   -0.4103    0.4643 H          1 UNK    0.0000
     57 H22          7.3818   -1.1550    2.0824 H          1 UNK    0.0000
     58 Cu1          3.0393   -0.5654   -0.1622 M.Cu       1 UNK    0.0000
@<TRIPOS>BOND
     1    1    2 1
     2    1    3 1
     3    1   16 1
     4    1   19 1
     5    2    4 1
     6    2    5 1
     7    2   14 1
     8    6    7 ar
     9    6   11 ar
    10    6   16 1
    11    7    8 ar
    12    7   12 1
    13    8    9 ar
    14    8   17 1
    15    9   10 ar
    16    9   13 1
    17   10   11 ar
    18   10   35 1
    19   11   14 1
    20   14   15 2
    21   15   36 1
    22   17   18 1
    23   19   20 ar
    24   19   21 ar
    25   20   22 ar
    26   20   23 1
    27   21   24 ar
    28   21   25 1
    29   22   26 ar
    30   22   28 1
    31   24   26 ar
    32   24   27 1
    33   26   30 1
    34   28   29 1
    35   30   31 1
    36   31   32 1
    37   31   33 1
    38   31   34 1
    39   36   37 1
    40   36   38 am
```

```
    41    38    39 2
    42    38    40 1
    43    40    41 ar
    44    40    42 ar
    45    41    43 ar
    46    41    44 1
    47    42    45 ar
    48    42    46 1
    49    43    47 ar
    50    43    48 1
    51    45    47 ar
    52    45    49 1
    53    47    50 1
    54    51    52 2
    55    51    53 2
    56    51    54 1
    57    54    55 1
    58    54    56 1
    59    54    57 1
    60    58    39 1
    61    58    15 1
    62    58    35 1
    63    58    53 1
@<TRIPOS>SUBSTRUCTURE
     1 UNK     1 RESIDUE           4 A     UNK     0 ROOT
```

Table S5.5. [Cu((*R*)-$L^1H_2^{am}$)(H_2O)]$^+$ ($CuC_{23}N_2H_{21}O_7^+$)

Electronic Energy, BS1 (a.u.)	=	-1722.54133998
Thermal and entropic correction, BS1 (a.u.)	=	0.348554
Electronic Energy, BS2 (a.u.)	=	-3166.3895989

```
@<TRIPOS>MOLECULE
CuC23N2H21O7
54 59 1 0 0
Cu_r-HHSB_wat
NO_CHARGES


@<TRIPOS>ATOM
      1 C1         -1.9850   -0.7649   -0.5372 C.3       1 UNK    0.0000
```

2	C2	-0.8245	0.0160	0.0697	C.3	1	UNK	0.0000
3	H1	-1.7669	-0.9585	-1.5951	H	1	UNK	0.0000
4	H2	-1.0520	0.2812	1.1102	H	1	UNK	0.0000
5	H3	-0.7098	0.9451	-0.4957	H	1	UNK	0.0000
6	C3	-0.9617	-2.8064	0.1260	C.ar	1	UNK	0.0000
7	C4	-1.1610	-4.1658	0.2655	C.ar	1	UNK	0.0000
8	C5	-0.0413	-5.0137	0.2738	C.ar	1	UNK	0.0000
9	C6	1.2451	-4.5107	0.1188	C.ar	1	UNK	0.0000
10	C7	1.4722	-3.1276	-0.0219	C.ar	1	UNK	0.0000
11	C8	0.3417	-2.2240	0.0065	C.ar	1	UNK	0.0000
12	H4	-2.1635	-4.5657	0.3532	H	1	UNK	0.0000
13	H5	2.1010	-5.1780	0.1032	H	1	UNK	0.0000
14	C9	0.4476	-0.7933	0.0314	C.2	1	UNK	0.0000
15	N1	1.6193	-0.2007	0.0649	N.pl3	1	UNK	0.0000
16	O1	-2.0950	-2.0403	0.1351	O.3	1	UNK	0.0000
17	O2	-0.2892	-6.3457	0.4198	O.3	1	UNK	0.0000
18	H6	0.5472	-6.8372	0.4064	H	1	UNK	0.0000
19	C10	-3.3028	-0.0468	-0.4110	C.ar	1	UNK	0.0000
20	C11	-3.9266	0.0694	0.8414	C.ar	1	UNK	0.0000
21	C12	-3.8888	0.5458	-1.5285	C.ar	1	UNK	0.0000
22	C13	-5.1211	0.7651	0.9643	C.ar	1	UNK	0.0000
23	H7	-3.4939	-0.3891	1.7246	H	1	UNK	0.0000
24	C14	-5.0871	1.2620	-1.4110	C.ar	1	UNK	0.0000
25	H8	-3.4149	0.4557	-2.5013	H	1	UNK	0.0000
26	C15	-5.7066	1.3745	-0.1686	C.ar	1	UNK	0.0000
27	H9	-5.5289	1.7186	-2.2884	H	1	UNK	0.0000
28	O3	-5.7256	0.8571	2.1943	O.3	1	UNK	0.0000
29	H10	-6.5494	1.3598	2.0854	H	1	UNK	0.0000
30	O4	-6.8780	2.0381	0.0900	O.3	1	UNK	0.0000
31	C16	-7.5428	2.6779	-1.0043	C.3	1	UNK	0.0000
32	H11	-7.8303	1.9497	-1.7710	H	1	UNK	0.0000
33	H12	-8.4374	3.1377	-0.5830	H	1	UNK	0.0000
34	H13	-6.9089	3.4512	-1.4527	H	1	UNK	0.0000
35	O5	2.7228	-2.7466	-0.1772	O.2cor	1	UNK	0.0000
36	N2	1.7007	1.1766	0.1557	N.am	1	UNK	0.0000
37	H14	0.9211	1.7112	0.5221	H	1	UNK	0.0000
38	C17	2.9195	1.7252	-0.0073	C.2	1	UNK	0.0000
39	O6	3.9111	0.9756	-0.2588	O.2cor	1	UNK	0.0000
40	C18	3.0730	3.1860	0.1183	C.ar	1	UNK	0.0000
41	C19	4.3448	3.6878	0.4396	C.ar	1	UNK	0.0000
42	C20	2.0006	4.0713	-0.0859	C.ar	1	UNK	0.0000
43	C21	4.5366	5.0603	0.5740	C.ar	1	UNK	0.0000
44	H15	5.1672	2.9976	0.5925	H	1	UNK	0.0000
45	C22	2.2027	5.4433	0.0459	C.ar	1	UNK	0.0000
46	H16	1.0212	3.7024	-0.3739	H	1	UNK	0.0000
47	C23	3.4668	5.9390	0.3789	C.ar	1	UNK	0.0000
48	H17	5.5185	5.4445	0.8316	H	1	UNK	0.0000
49	H18	1.3754	6.1259	-0.1199	H	1	UNK	0.0000
50	H19	3.6189	7.0092	0.4811	H	1	UNK	0.0000

```
     51 Cu1          3.4034   -0.9714   -0.2334 M.Cu       1 UNK    0.0000
     52 O7           5.3075   -1.6690   -0.4492 O.H2O      1 UNK    0.0000
     53 H20          5.7476   -1.2004   -1.1767 H          1 UNK    0.0000
     54 H21          5.2618   -2.5973   -0.7306 H          1 UNK    0.0000
@<TRIPOS>BOND
     1    1    2 1
     2    1    3 1
     3    1   16 1
     4    1   19 1
     5    2    4 1
     6    2    5 1
     7    2   14 1
     8    6    7 ar
     9    6   11 ar
    10    6   16 1
    11    7    8 ar
    12    7   12 1
    13    8    9 ar
    14    8   17 1
    15    9   10 ar
    16    9   13 1
    17   10   11 ar
    18   10   35 1
    19   11   14 1
    20   14   15 2
    21   15   36 1
    22   17   18 1
    23   19   20 ar
    24   19   21 ar
    25   20   22 ar
    26   20   23 1
    27   21   24 ar
    28   21   25 1
    29   22   26 ar
    30   22   28 1
    31   24   26 ar
    32   24   27 1
    33   26   30 1
    34   28   29 1
    35   30   31 1
    36   31   32 1
    37   31   33 1
    38   31   34 1
    39   36   37 1
    40   36   38 am
    41   38   39 2
    42   38   40 1
    43   40   41 ar
    44   40   42 ar
```

```
    45   41   43 ar
    46   41   44 1
    47   42   45 ar
    48   42   46 1
    49   43   47 ar
    50   43   48 1
    51   45   47 ar
    52   45   49 1
    53   47   50 1
    54   52   53 1
    55   52   54 1
    56   51   39 1
    57   51   35 1
    58   51   52 1
    59   51   15 1
@<TRIPOS>SUBSTRUCTURE
     1 UNK      1 RESIDUE           4 A      UNK      0 ROOT
```

Table S5.6. [Cu((*S*)-$L^1H_2^{am}$)(H_2O)]$^+$ ($CuC_{23}N_2H_{21}O_7^+$)

Electronic Energy, BS1 (a.u.)	=	-1722.54133998
Thermal and entropic correction, BS1 (a.u.)	=	0.348554
Electronic Energy, BS2 (a.u.)	=	-3166.3895989

```
@<TRIPOS>MOLECULE
CuC23N2H21O7
54 59 1 0 0
Cu_s-HHSB_wat
NO_CHARGES


@<TRIPOS>ATOM
      1 C1          -1.9850   -0.7649    0.5372 C.3       1 UNK     0.0000
      2 C2          -0.8245    0.0160   -0.0697 C.3       1 UNK     0.0000
      3 H1          -1.7669   -0.9585    1.5951 H         1 UNK     0.0000
      4 H2          -1.0520    0.2812   -1.1102 H         1 UNK     0.0000
      5 H3          -0.7098    0.9451    0.4957 H         1 UNK     0.0000
      6 C3          -0.9617   -2.8064   -0.1260 C.ar      1 UNK     0.0000
      7 C4          -1.1610   -4.1658   -0.2655 C.ar      1 UNK     0.0000
      8 C5          -0.0413   -5.0137   -0.2738 C.ar      1 UNK     0.0000
      9 C6           1.2451   -4.5107   -0.1188 C.ar      1 UNK     0.0000
```

```
    10 C7          1.4722   -3.1276    0.0219 C.ar      1 UNK    0.0000
    11 C8          0.3417   -2.2240   -0.0065 C.ar      1 UNK    0.0000
    12 H4         -2.1635   -4.5657   -0.3532 H         1 UNK    0.0000
    13 H5          2.1010   -5.1780   -0.1032 H         1 UNK    0.0000
    14 C9          0.4476   -0.7933   -0.0314 C.2       1 UNK    0.0000
    15 N1          1.6193   -0.2007   -0.0649 N.pl3     1 UNK    0.0000
    16 O1         -2.0950   -2.0403   -0.1351 O.3       1 UNK    0.0000
    17 O2         -0.2892   -6.3457   -0.4198 O.3       1 UNK    0.0000
    18 H6          0.5472   -6.8372   -0.4064 H         1 UNK    0.0000
    19 C10        -3.3028   -0.0468    0.4110 C.ar      1 UNK    0.0000
    20 C11        -3.9266    0.0694   -0.8414 C.ar      1 UNK    0.0000
    21 C12        -3.8888    0.5458    1.5285 C.ar      1 UNK    0.0000
    22 C13        -5.1211    0.7651   -0.9643 C.ar      1 UNK    0.0000
    23 H7         -3.4939   -0.3891   -1.7246 H         1 UNK    0.0000
    24 C14        -5.0871    1.2620    1.4110 C.ar      1 UNK    0.0000
    25 H8         -3.4149    0.4557    2.5013 H         1 UNK    0.0000
    26 C15        -5.7066    1.3745    0.1686 C.ar      1 UNK    0.0000
    27 H9         -5.5289    1.7186    2.2884 H         1 UNK    0.0000
    28 O3         -5.7256    0.8571   -2.1943 O.3       1 UNK    0.0000
    29 H10        -6.5494    1.3598   -2.0854 H         1 UNK    0.0000
    30 O4         -6.8780    2.0381   -0.0900 O.3       1 UNK    0.0000
    31 C16        -7.5428    2.6779    1.0043 C.3       1 UNK    0.0000
    32 H11        -7.8303    1.9497    1.7710 H         1 UNK    0.0000
    33 H12        -8.4374    3.1377    0.5830 H         1 UNK    0.0000
    34 H13        -6.9089    3.4512    1.4527 H         1 UNK    0.0000
    35 O5          2.7228   -2.7466    0.1772 O.2cor    1 UNK    0.0000
    36 N2          1.7007    1.1766   -0.1557 N.am      1 UNK    0.0000
    37 H14         0.9211    1.7112   -0.5221 H         1 UNK    0.0000
    38 C17         2.9195    1.7252    0.0073 C.2       1 UNK    0.0000
    39 O6          3.9112    0.9756    0.2588 O.2cor    1 UNK    0.0000
    40 C18         3.0730    3.1860   -0.1183 C.ar      1 UNK    0.0000
    41 C19         4.3448    3.6878   -0.4396 C.ar      1 UNK    0.0000
    42 C20         2.0006    4.0713    0.0859 C.ar      1 UNK    0.0000
    43 C21         4.5366    5.0603   -0.5740 C.ar      1 UNK    0.0000
    44 H15         5.1672    2.9976   -0.5925 H         1 UNK    0.0000
    45 C22         2.2027    5.4433   -0.0459 C.ar      1 UNK    0.0000
    46 H16         1.0212    3.7024    0.3739 H         1 UNK    0.0000
    47 C23         3.4668    5.9390   -0.3789 C.ar      1 UNK    0.0000
    48 H17         5.5185    5.4445   -0.8316 H         1 UNK    0.0000
    49 H18         1.3754    6.1259    0.1199 H         1 UNK    0.0000
    50 H19         3.6189    7.0092   -0.4811 H         1 UNK    0.0000
    51 Cu1         3.4034   -0.9714    0.2334 M.Cu      1 UNK    0.0000
    52 O7          5.3075   -1.6690    0.4492 O.H2O     1 UNK    0.0000
    53 H20         5.7476   -1.2004    1.1767 H         1 UNK    0.0000
    54 H21         5.2618   -2.5973    0.7306 H         1 UNK    0.0000
@<TRIPOS>BOND
     1    1    2 1
     2    1    3 1
     3    1   16 1
```

4	1	19	1
5	2	4	1
6	2	5	1
7	2	14	1
8	6	7	ar
9	6	11	ar
10	6	16	1
11	7	8	ar
12	7	12	1
13	8	9	ar
14	8	17	1
15	9	10	ar
16	9	13	1
17	10	11	ar
18	10	35	1
19	11	14	1
20	14	15	2
21	15	36	1
22	17	18	1
23	19	20	ar
24	19	21	ar
25	20	22	ar
26	20	23	1
27	21	24	ar
28	21	25	1
29	22	26	ar
30	22	28	1
31	24	26	ar
32	24	27	1
33	26	30	1
34	28	29	1
35	30	31	1
36	31	32	1
37	31	33	1
38	31	34	1
39	36	37	1
40	36	38	am
41	38	39	2
42	38	40	1
43	40	41	ar
44	40	42	ar
45	41	43	ar
46	41	44	1
47	42	45	ar
48	42	46	1
49	43	47	ar
50	43	48	1
51	45	47	ar
52	45	49	1

```
    53   47   50 1
    54   52   53 1
    55   52   54 1
    56   51   35 1
    57   51   52 1
    58   51   15 1
    59   51   39 1
@<TRIPOS>SUBSTRUCTURE
     1 UNK     1 RESIDUE           4 A     UNK     0 ROOT
```

Table S5.7. [Cu((*R*)-$L^2H_2^{am}$)(AcO)] (CuHIN, $CuC_{24}N_3H_{21}O_8$)

Electronic Energy, BS1 (a.u.) -1890.78523384

Thermal and entropic correction, BS1 (a.u.) 0.356107

```
@<TRIPOS>MOLECULE
CuC24N3H21O8
57 62 1 0 0
CuHIN
NO_CHARGES


@<TRIPOS>ATOM
      1 C1          2.3494   -0.9091    0.5524 C.3       1 UNK    0.0000
      2 C2          1.2881    0.0074   -0.0450 C.3       1 UNK    0.0000
      3 H1          2.0849   -1.1282    1.5945 H         1 UNK    0.0000
      4 H2          1.5907    0.3321   -1.0491 H         1 UNK    0.0000
      5 H3          1.2264    0.8968    0.5893 H         1 UNK    0.0000
      6 C3          1.1628   -2.8133   -0.2357 C.ar      1 UNK    0.0000
      7 C4          1.2427   -4.1817   -0.3954 C.ar      1 UNK    0.0000
      8 C5          0.0516   -4.9215   -0.4866 C.ar      1 UNK    0.0000
      9 C6         -1.1886   -4.3023   -0.4067 C.ar      1 UNK    0.0000
     10 C7         -1.2962   -2.9038   -0.2504 C.ar      1 UNK    0.0000
     11 C8         -0.0853   -2.1115   -0.1714 C.ar      1 UNK    0.0000
     12 H4          2.2072   -4.6725   -0.4358 H         1 UNK    0.0000
     13 H5         -2.1027   -4.8847   -0.4642 H         1 UNK    0.0000
     14 C9         -0.0537   -0.6779   -0.1360 C.2       1 UNK    0.0000
     15 N1         -1.1589    0.0300   -0.2262 N.pl3     1 UNK    0.0000
     16 O1          2.3629   -2.1583   -0.1752 O.3       1 UNK    0.0000
     17 O2          0.1882   -6.2691   -0.6393 O.3       1 UNK    0.0000
     18 H6         -0.6874   -6.6850   -0.6657 H         1 UNK    0.0000
     19 C10         3.7336   -0.3181    0.4874 C.ar      1 UNK    0.0000
     20 C11         4.3682   -0.1569   -0.7549 C.ar      1 UNK    0.0000
     21 C12         4.3802    0.1020    1.6485 C.ar      1 UNK    0.0000
     22 C13         5.6310    0.4131   -0.8256 C.ar      1 UNK    0.0000
     23 H7          3.8889   -0.4821   -1.6727 H         1 UNK    0.0000
     24 C14         5.6514    0.6886    1.5857 C.ar      1 UNK    0.0000
```

```
     25 H8           3.8977   -0.0240    2.6131 H          1 UNK     0.0000
     26 C15          6.2805    0.8450    0.3530 C.ar       1 UNK     0.0000
     27 H9           6.1414    1.0105    2.4967 H          1 UNK     0.0000
     28 O3           6.2418    0.5553   -2.0477 O.3        1 UNK     0.0000
     29 H10          7.1082    0.9691   -1.9008 H          1 UNK     0.0000
     30 O4           7.5198    1.3927    0.1447 O.3        1 UNK     0.0000
     31 C16          8.2472    1.8580    1.2863 C.3        1 UNK     0.0000
     32 H11          8.4513    1.0383    1.9843 H          1 UNK     0.0000
     33 H12          9.1881    2.2526    0.9010 H          1 UNK     0.0000
     34 H13          7.6999    2.6534    1.8045 H          1 UNK     0.0000
     35 O5          -2.5100   -2.4087   -0.1815 O.2cor     1 UNK     0.0000
     36 N2          -1.0859    1.4104   -0.2664 N.pl3      1 UNK     0.0000
     37 H14         -0.2154    1.8783   -0.4960 H          1 UNK     0.0000
     38 C17         -2.2494    2.0814   -0.2456 C.2        1 UNK     0.0000
     39 O6          -3.3434    1.4565   -0.1720 O.2cor     1 UNK     0.0000
     40 C18         -2.2116    3.5607   -0.3128 C.ar       1 UNK     0.0000
     41 C19         -3.3224    4.2385   -0.8259 C.ar       1 UNK     0.0000
     42 C20         -1.1195    4.3096    0.1404 C.ar       1 UNK     0.0000
     43 C21         -3.2814    5.6290   -0.8812 C.ar       1 UNK     0.0000
     44 H15         -4.1900    3.6940   -1.1799 H          1 UNK     0.0000
     45 C22         -1.1867    5.6980    0.0433 C.ar       1 UNK     0.0000
     46 H16         -0.2468    3.8424    0.5838 H          1 UNK     0.0000
     47 H17         -4.1286    6.1776   -1.2840 H          1 UNK     0.0000
     48 H18         -0.3535    6.3013    0.3935 H          1 UNK     0.0000
     49 C23         -5.5086   -0.8982    1.0390 C.2        1 UNK     0.0000
     50 O7          -4.8906   -0.6437    2.0921 O.2        1 UNK     0.0000
     51 O8          -4.9531   -0.9667   -0.1346 O.2cor     1 UNK     0.0000
     52 C24         -7.0040   -1.1519    1.0500 C.3        1 UNK     0.0000
     53 H19         -7.2167   -2.1276    0.6027 H          1 UNK     0.0000
     54 H20         -7.5064   -0.3957    0.4384 H          1 UNK     0.0000
     55 H21         -7.3968   -1.1187    2.0672 H          1 UNK     0.0000
     56 Cu1         -3.0434   -0.5630   -0.1595 M.Cu       1 UNK     0.0000
     57 N3          -2.2387    6.3635   -0.4594 N.ar       1 UNK     0.0000
@<TRIPOS>BOND
     1     1     2 1
     2     1     3 1
     3     1    16 1
     4     1    19 1
     5     2     4 1
     6     2     5 1
     7     2    14 1
     8     6     7 ar
     9     6    11 ar
    10     6    16 1
    11     7     8 ar
    12     7    12 1
    13     8     9 ar
    14     8    17 1
    15     9    10 ar
    16     9    13 1
    17    10    11 ar
    18    10    35 1
    19    11    14 1
    20    14    15 2
    21    15    36 1
    22    17    18 1
    23    19    20 ar
```

```
    24    19    21 ar
    25    20    22 ar
    26    20    23 1
    27    21    24 ar
    28    21    25 1
    29    22    26 ar
    30    22    28 1
    31    24    26 ar
    32    24    27 1
    33    26    30 1
    34    28    29 1
    35    30    31 1
    36    31    32 1
    37    31    33 1
    38    31    34 1
    39    36    37 1
    40    36    38 am
    41    38    39 2
    42    38    40 1
    43    40    41 ar
    44    40    42 ar
    45    41    43 ar
    46    41    44 1
    47    42    45 ar
    48    42    46 1
    49    43    47 1
    50    43    57 ar
    51    45    48 1
    52    45    57 ar
    53    49    50 2
    54    49    51 2
    55    49    52 1
    56    52    53 1
    57    52    54 1
    58    52    55 1
    59    56    51 1
    60    56    35 1
    61    56    15 1
    62    56    39 1
@<TRIPOS>SUBSTRUCTURE
     1 UNK      1 RESIDUE           4 A     UNK     0 ROOT
```

Table S5.8. [Cu((*R*)-$L^2H_2^{am}$)(H_2O)]$^+$ ($CuC_{22}N_3H_{20}O_7^+$)

Electronic Energy, BS1 (a.u.) -1738.57510149

Thermal and entropic correction, BS1 (a.u.) 0.337744

```
@<TRIPOS>MOLECULE
```

```
CuC22N3H20O7
53 58 1 0 0
CuHIN_wat
NO_CHARGES


@<TRIPOS>ATOM
      1 C1          1.9869    0.7671   -0.5370 C.3       1 UNK    0.0000
      2 C2          0.8259   -0.0165    0.0655 C.3       1 UNK    0.0000
      3 H1          1.7703    0.9644   -1.5945 H         1 UNK    0.0000
      4 H2          1.0518   -0.2862    1.1052 H         1 UNK    0.0000
      5 H3          0.7136   -0.9427   -0.5051 H         1 UNK    0.0000
      6 C3          0.9615    2.8055    0.1304 C.ar      1 UNK    0.0000
      7 C4          1.1584    4.1641    0.2745 C.ar      1 UNK    0.0000
      8 C5          0.0374    5.0111    0.2823 C.ar      1 UNK    0.0000
      9 C6         -1.2482    4.5077    0.1222 C.ar      1 UNK    0.0000
     10 C7         -1.4739    3.1251   -0.0234 C.ar      1 UNK    0.0000
     11 C8         -0.3421    2.2218    0.0065 C.ar      1 UNK    0.0000
     12 H4          2.1601    4.5653    0.3662 H         1 UNK    0.0000
     13 H5         -2.1043    5.1746    0.1057 H         1 UNK    0.0000
     14 C9         -0.4464    0.7929    0.0298 C.2       1 UNK    0.0000
     15 N1         -1.6196    0.1999    0.0645 N.pl3     1 UNK    0.0000
     16 O1          2.0948    2.0404    0.1400 O.3       1 UNK    0.0000
     17 O2          0.2841    6.3420    0.4327 O.3       1 UNK    0.0000
     18 H6         -0.5522    6.8338    0.4179 H         1 UNK    0.0000
     19 C10         3.3048    0.0494   -0.4113 C.ar      1 UNK    0.0000
     20 C11         3.9267   -0.0714    0.8417 C.ar      1 UNK    0.0000
     21 C12         3.8930   -0.5381   -1.5303 C.ar      1 UNK    0.0000
     22 C13         5.1213   -0.7669    0.9637 C.ar      1 UNK    0.0000
     23 H7          3.4921    0.3831    1.7261 H         1 UNK    0.0000
     24 C14         5.0916   -1.2540   -1.4138 C.ar      1 UNK    0.0000
     25 H8          3.4207   -0.4444   -2.5035 H         1 UNK    0.0000
     26 C15         5.7091   -1.3713   -0.1708 C.ar      1 UNK    0.0000
     27 H9          5.5352   -1.7066   -2.2923 H         1 UNK    0.0000
     28 O3          5.7237   -0.8636    2.1943 O.3       1 UNK    0.0000
     29 H10         6.5482   -1.3650    2.0849 H         1 UNK    0.0000
     30 O4          6.8804   -2.0350    0.0869 O.3       1 UNK    0.0000
     31 C16         7.5468   -2.6711   -1.0087 C.3       1 UNK    0.0000
     32 H11         7.8357   -1.9402   -1.7723 H         1 UNK    0.0000
     33 H12         8.4407   -3.1326   -0.5876 H         1 UNK    0.0000
     34 H13         6.9135   -3.4428   -1.4607 H         1 UNK    0.0000
     35 O5         -2.7229    2.7441   -0.1857 O.2cor    1 UNK    0.0000
     36 N2         -1.6997   -1.1770    0.1527 N.am      1 UNK    0.0000
     37 H14        -0.9093   -1.7183    0.4862 H         1 UNK    0.0000
     38 C17        -2.9159   -1.7221   -0.0058 C.2       1 UNK    0.0000
     39 O6         -3.9131   -0.9837   -0.2536 O.2cor    1 UNK    0.0000
     40 C18        -3.0684   -3.1894    0.1247 C.ar      1 UNK    0.0000
     41 C19        -4.3257   -3.7007    0.4644 C.ar      1 UNK    0.0000
     42 C20        -2.0172   -4.0878   -0.0938 C.ar      1 UNK    0.0000
```

```
     43 C21         -4.4692   -5.0800    0.5867 C.ar       1 UNK    0.0000
     44 H15         -5.1657   -3.0384    0.6376 H          1 UNK    0.0000
     45 C22         -2.2733   -5.4496    0.0513 C.ar       1 UNK    0.0000
     46 H16         -1.0280   -3.7583   -0.3929 H          1 UNK    0.0000
     47 H17         -5.4343   -5.4994    0.8576 H          1 UNK    0.0000
     48 H18         -1.4741   -6.1662   -0.1176 H          1 UNK    0.0000
     49 Cu1         -3.4059    0.9700   -0.2385 M.Cu       1 UNK    0.0000
     50 N3          -3.4702   -5.9556    0.3881 N.ar       1 UNK    0.0000
     51 O7          -5.3130    1.6541   -0.4643 O.H2O      1 UNK    0.0000
     52 H19         -5.7981    1.0633   -1.0629 H          1 UNK    0.0000
     53 H20         -5.2913    2.5141   -0.9143 H          1 UNK    0.0000
@<TRIPOS>BOND
     1    1    2 1
     2    1    3 1
     3    1   16 1
     4    1   19 1
     5    2    4 1
     6    2    5 1
     7    2   14 1
     8    6    7 ar
     9    6   11 ar
    10    6   16 1
    11    7    8 ar
    12    7   12 1
    13    8    9 ar
    14    8   17 1
    15    9   10 ar
    16    9   13 1
    17   10   11 ar
    18   10   35 1
    19   11   14 1
    20   14   15 2
    21   15   36 1
    22   17   18 1
    23   19   20 ar
    24   19   21 ar
    25   20   22 ar
    26   20   23 1
    27   21   24 ar
    28   21   25 1
    29   22   26 ar
    30   22   28 1
    31   24   26 ar
    32   24   27 1
    33   26   30 1
    34   28   29 1
    35   30   31 1
    36   31   32 1
    37   31   33 1
```

```
    38   31   34 1
    39   36   37 1
    40   36   38 am
    41   38   39 2
    42   38   40 1
    43   40   41 ar
    44   40   42 ar
    45   41   43 ar
    46   41   44 1
    47   42   45 ar
    48   42   46 1
    49   43   47 1
    50   43   50 ar
    51   45   48 1
    52   45   50 ar
    53   51   52 1
    54   51   53 1
    55   49   51 1
    56   49   35 1
    57   49   15 1
    58   49   39 1
@<TRIPOS>SUBSTRUCTURE
     1 UNK      1 RESIDUE           4 A      UNK      0 ROOT
```

Table S5.9. [Cu((*R*)-L^3H_2-κS^{am})(AcO)] (CuHTSC; $CuC_{19}N_3H_{19}SO_7$)

Electronic Energy, BS1 (a.u.) -2022.01285334

Thermal and entropic correction, BS1 (a.u.) 0.305947

```
@<TRIPOS>MOLECULE
CuC19N3H19SO7
50 54 1 0 0
CuHTSC
NO_CHARGES


@<TRIPOS>ATOM
      1 C1          -1.9790    0.4760    0.4765 C.3       1 UNK     0.0000
      2 C2          -1.1524   -0.4620   -0.3961 C.3       1 UNK     0.0000
      3 H1          -1.5826    0.4496    1.4998 H         1 UNK     0.0000
      4 H2          -1.5704   -0.4920   -1.4108 H         1 UNK     0.0000
      5 H3          -1.2293   -1.4662    0.0282 H         1 UNK     0.0000
      6 C3          -0.5636    2.2790   -0.1644 C.ar      1 UNK     0.0000
```

```
     7 C4          -0.4307    3.6541   -0.1796 C.ar      1 UNK     0.0000
     8 C5           0.8495    4.2077   -0.3414 C.ar      1 UNK     0.0000
     9 C6           1.9716    3.4000   -0.4667 C.ar      1 UNK     0.0000
    10 C7           1.8599    1.9929   -0.4485 C.ar      1 UNK     0.0000
    11 C8           0.5505    1.3900   -0.3136 C.ar      1 UNK     0.0000
    12 H4          -1.2999    4.2878   -0.0541 H         1 UNK     0.0000
    13 H5           2.9598    3.8365   -0.5733 H         1 UNK     0.0000
    14 C9           0.2906   -0.0164   -0.4553 C.2       1 UNK     0.0000
    15 N1           1.2534   -0.8929   -0.6698 N.pl3     1 UNK     0.0000
    16 O1          -1.8453    1.8242   -0.0201 O.3       1 UNK     0.0000
    17 O2           0.9248    5.5702   -0.3444 O.3       1 UNK     0.0000
    18 H6           1.8504    5.8431   -0.4403 H         1 UNK     0.0000
    19 C10         -3.4441    0.1238    0.4862 C.ar      1 UNK     0.0000
    20 C11         -4.2305    0.3562   -0.6534 C.ar      1 UNK     0.0000
    21 C12         -4.0194   -0.4705    1.6082 C.ar      1 UNK     0.0000
    22 C13         -5.5718    0.0007   -0.6605 C.ar      1 UNK     0.0000
    23 H7          -3.8074    0.8230   -1.5369 H         1 UNK     0.0000
    24 C14         -5.3703   -0.8426    1.6059 C.ar      1 UNK     0.0000
    25 H8          -3.4188   -0.6495    2.4950 H         1 UNK     0.0000
    26 C15         -6.1502   -0.6081    0.4759 C.ar      1 UNK     0.0000
    27 H9          -5.8021   -1.3035    2.4862 H         1 UNK     0.0000
    28 O3          -6.3299    0.2436   -1.7801 O.3       1 UNK     0.0000
    29 H10         -7.2303   -0.0752   -1.6047 H         1 UNK     0.0000
    30 O4          -7.4798   -0.9122    0.3362 O.3       1 UNK     0.0000
    31 C16         -8.1452   -1.5383    1.4381 C.3       1 UNK     0.0000
    32 H11         -8.1309   -0.8942    2.3246 H         1 UNK     0.0000
    33 H12         -9.1755   -1.6936    1.1162 H         1 UNK     0.0000
    34 H13         -7.6858   -2.5038    1.6784 H         1 UNK     0.0000
    35 O5           2.9668    1.3041   -0.5718 O.2cor    1 UNK     0.0000
    36 N2           0.8712   -2.2007   -0.9402 N.pl3     1 UNK     0.0000
    37 H14         -0.0128   -2.3571   -1.4159 H         1 UNK     0.0000
    38 C17          1.7498   -3.2152   -0.8511 C.2       1 UNK     0.0000
    39 C18          5.3134   -0.1827    1.4012 C.2       1 UNK     0.0000
    40 O6           4.4235   -0.1480    2.2752 O.2       1 UNK     0.0000
    41 O7           5.0898   -0.3731    0.1362 O.2cor    1 UNK     0.0000
    42 C19          6.7753   -0.0077    1.7687 C.3       1 UNK     0.0000
    43 H15          7.2034    0.8231    1.1995 H         1 UNK     0.0000
    44 H16          7.3301   -0.9111    1.4956 H         1 UNK     0.0000
    45 H17          6.8879    0.1810    2.8372 H         1 UNK     0.0000
    46 Cu1          3.1954   -0.6024   -0.3155 M.Cu      1 UNK     0.0000
    47 N3           1.3214   -4.4278   -1.2054 N.pl3     1 UNK     0.0000
    48 H18          0.3595   -4.5924   -1.4784 H         1 UNK     0.0000
    49 H19          1.9465   -5.2190   -1.1493 H         1 UNK     0.0000
    50 S1           3.3624   -2.9536   -0.3043 S.a       1 UNK     0.0000
@<TRIPOS>BOND
     1     1     2 1
     2     1     3 1
     3     1    16 1
     4     1    19 1
     5     2     4 1
     6     2     5 1
     7     2    14 1
     8     6     7 ar
     9     6    11 ar
    10     6    16 1
    11     7     8 ar
    12     7    12 1
```

```
    13     8     9 ar
    14     8    17 1
    15     9    10 ar
    16     9    13 1
    17    10    11 ar
    18    10    35 1
    19    11    14 1
    20    14    15 2
    21    15    36 2
    22    17    18 1
    23    19    20 ar
    24    19    21 ar
    25    20    22 ar
    26    20    23 1
    27    21    24 ar
    28    21    25 1
    29    22    26 ar
    30    22    28 1
    31    24    26 ar
    32    24    27 1
    33    26    30 1
    34    28    29 1
    35    30    31 1
    36    31    32 1
    37    31    33 1
    38    31    34 1
    39    36    37 1
    40    36    38 2
    41    38    47 2
    42    38    50 2
    43    39    40 2
    44    39    41 2
    45    39    42 1
    46    42    43 1
    47    42    44 1
    48    42    45 1
    49    47    48 1
    50    47    49 1
    51    46    35 1
    52    46    15 1
    53    46    41 1
    54    46    50 1
@<TRIPOS>SUBSTRUCTURE
     1 UNK      1 RESIDUE           4 A      UNK      0 ROOT
```

Table S5.10. [Cu((*R*)-L^3H_2-κS^{am})(H_2O)]$^+$ ($CuC_{17}N_3H_{18}SO_6^+$)

Electronic Energy, BS1 (a.u.)	-1869.80106706
Thermal and entropic correction, BS1 (a.u.)	0.287400

```
@<TRIPOS>MOLECULE
CuC17N3H18SO6
46 50 1 0 0
CuHTSC_wat
NO_CHARGES


@<TRIPOS>ATOM
      1 C1          1.5402    0.4993   -0.5857 C.3       1 UNK     0.0000
      2 C2          0.6394   -0.4733    0.1666 C.3       1 UNK     0.0000
      3 H1          1.2525    0.5065   -1.6446 H         1 UNK     0.0000
      4 H2          0.9729   -0.5590    1.2092 H         1 UNK     0.0000
      5 H3          0.7465   -1.4559   -0.3004 H         1 UNK     0.0000
      6 C3          0.0564    2.2758   -0.0275 C.ar      1 UNK     0.0000
      7 C4         -0.0895    3.6488    0.0021 C.ar      1 UNK     0.0000
      8 C5         -1.3832    4.1911    0.0633 C.ar      1 UNK     0.0000
      9 C6         -2.5065    3.3736    0.0752 C.ar      1 UNK     0.0000
     10 C7         -2.3800    1.9702    0.0437 C.ar      1 UNK     0.0000
     11 C8         -1.0609    1.3778    0.0074 C.ar      1 UNK     0.0000
     12 H4          0.7819    4.2909   -0.0310 H         1 UNK     0.0000
     13 H5         -3.5035    3.8013    0.1054 H         1 UNK     0.0000
     14 C9         -0.8027   -0.0272    0.1394 C.2       1 UNK     0.0000
     15 N1         -1.7710   -0.9121    0.2862 N.pl.3    1 UNK     0.0000
     16 O1          1.3475    1.8303   -0.0612 O.3       1 UNK     0.0000
     17 O2         -1.4686    5.5510    0.0896 O.3       1 UNK     0.0000
     18 H6         -2.4001    5.8194    0.1176 H         1 UNK     0.0000
     19 C10         3.0001    0.1508   -0.4516 C.ar      1 UNK     0.0000
     20 C11         3.6579    0.3467    0.7733 C.ar      1 UNK     0.0000
     21 C12         3.6937   -0.4112   -1.5220 C.ar      1 UNK     0.0000
     22 C13         4.9906   -0.0121    0.9157 C.ar      1 UNK     0.0000
     23 H7          3.1410    0.7876    1.6195 H         1 UNK     0.0000
     24 C14         5.0364   -0.7875   -1.3837 C.ar      1 UNK     0.0000
     25 H8          3.1922   -0.5626   -2.4732 H         1 UNK     0.0000
     26 C15         5.6889   -0.5895   -0.1690 C.ar      1 UNK     0.0000
     27 H9          5.5616   -1.2247   -2.2244 H         1 UNK     0.0000
     28 O3          5.6207    0.1952    2.1187 O.3       1 UNK     0.0000
     29 H10         6.5385   -0.1098    2.0319 H         1 UNK     0.0000
     30 O4          6.9950   -0.9033    0.1044 O.3       1 UNK     0.0000
     31 C16         7.7758   -1.4968   -0.9379 C.3       1 UNK     0.0000
     32 H11         7.8549   -0.8267   -1.8013 H         1 UNK     0.0000
     33 H12         8.7661   -1.6627   -0.5122 H         1 UNK     0.0000
     34 H13         7.3471   -2.4544   -1.2545 H         1 UNK     0.0000
     35 O5         -3.4926    1.2731    0.0601 O.2cor    1 UNK     0.0000
     36 N2         -1.4033   -2.2241    0.5552 N.pl3     1 UNK     0.0000
     37 H14        -0.5457   -2.3884    1.0766 H         1 UNK     0.0000
     38 C17        -2.2774   -3.2334    0.4042 C.2       1 UNK     0.0000
     39 Cu1        -3.6880   -0.6209   -0.1075 M.Cu      1 UNK     0.0000
     40 N3         -1.8781   -4.4546    0.7552 N.pl3     1 UNK     0.0000
```

```
     41 H15         -0.9346   -4.6303    1.0815 H          1 UNK     0.0000
     42 H16         -2.5087   -5.2387    0.6657 H          1 UNK     0.0000
     43 S1          -3.8618   -2.9585   -0.2254 S.a        1 UNK     0.0000
     44 O6          -5.6911   -0.3250   -0.5158 O.H2O      1 UNK     0.0000
     45 H17         -6.2670   -0.9860   -0.0996 H          1 UNK     0.0000
     46 H18         -5.8507   -0.4162   -1.4696 H          1 UNK     0.0000
@<TRIPOS>BOND
     1    1    2 1
     2    1    3 1
     3    1   16 1
     4    1   19 1
     5    2    4 1
     6    2    5 1
     7    2   14 1
     8    6    7 ar
     9    6   11 ar
    10    6   16 1
    11    7    8 ar
    12    7   12 1
    13    8    9 ar
    14    8   17 1
    15    9   10 ar
    16    9   13 1
    17   10   11 ar
    18   10   35 1
    19   11   14 1
    20   14   15 2
    21   15   36 2
    22   17   18 1
    23   19   20 ar
    24   19   21 ar
    25   20   22 ar
    26   20   23 1
    27   21   24 ar
    28   21   25 1
    29   22   26 ar
    30   22   28 1
    31   24   26 ar
    32   24   27 1
    33   26   30 1
    34   28   29 1
    35   30   31 1
    36   31   32 1
    37   31   33 1
    38   31   34 1
    39   36   37 1
    40   36   38 2
    41   38   40 2
    42   38   43 2
```

```
    43   40   41 1
    44   40   42 1
    45   44   45 1
    46   44   46 1
    47   39   15 1
    48   39   43 1
    49   39   44 1
    50   39   35 1
@<TRIPOS>SUBSTRUCTURE
     1 UNK     1 RESIDUE          4 A     UNK     0 ROOT
```

Table S5.11. TO (TO$^+$; $C_{19}N_2H_{17}S^+$)

Electronic Energy, BS1 (a.u.) -1241.87416378

Thermal and entropic correction, BS1 (a.u.) 0.271283

```
@<TRIPOS>MOLECULE
C19N2H17S
39 42 1 0 0
thiazole_orange
NO_CHARGES


@<TRIPOS>ATOM
      1 C1         -1.5760    4.7040   -5.8520 C.ar      1 UNK    0.0000
      2 C2         -2.7120    4.7390   -6.6710 C.ar      1 UNK    0.0000
      3 C3         -3.9770    4.8810   -6.0870 C.ar      1 UNK    0.0000
      4 C4         -4.0990    4.9340   -4.6900 C.ar      1 UNK    0.0000
      5 N1         -2.9330    4.9120   -2.4620 N.pl3     1 UNK    0.0000
      6 C5         -4.1310    4.7790   -1.5930 C.3       1 UNK    0.0000
      7 C6         -1.6450    5.0440   -2.0460 C.ar      1 UNK    0.0000
      8 C7         -1.3260    5.1750   -0.6170 C.2       1 UNK    0.0000
      9 S1         -0.5170    5.0450   -3.2500 S.a       1 UNK    0.0000
     10 C8         -1.6980    4.8620   -4.4530 C.ar      1 UNK    0.0000
     11 C9         -2.9600    4.9190   -3.8490 C.ar      1 UNK    0.0000
     12 C10         1.1580    5.7840    2.1560 C.ar      1 UNK    0.0000
     13 C11         1.2310    6.4260    3.4040 C.ar      1 UNK    0.0000
     14 C12         0.0860    6.6360    4.1640 C.ar      1 UNK    0.0000
     15 C13        -1.1580    6.3060    3.6230 C.ar      1 UNK    0.0000
     16 C14        -1.2600    5.8950    2.2780 C.ar      1 UNK    0.0000
     17 C15        -0.0930    5.6360    1.5050 C.ar      1 UNK    0.0000
     18 C16        -0.1140    5.2340    0.1300 C.ar      1 UNK    0.0000
```

```
     19 C17          1.1180    4.8860   -0.4700 C.ar       1 UNK    0.0000
     20 C18          2.3090    4.8990    0.2650 C.ar       1 UNK    0.0000
     21 N2           2.3190    5.3070    1.5670 N.pl3      1 UNK    0.0000
     22 C19          3.6210    5.2150    2.2580 C.3        1 UNK    0.0000
     23 H1          -0.6140    4.5500   -6.3150 H          1 UNK    0.0000
     24 H2          -2.6100    4.6600   -7.7440 H          1 UNK    0.0000
     25 H3          -4.8580    4.9500   -6.7060 H          1 UNK    0.0000
     26 H4          -5.0910    4.9950   -4.2700 H          1 UNK    0.0000
     27 H5          -4.9790    4.3900   -2.1530 H          1 UNK    0.0000
     28 H6          -3.9360    4.0720   -0.7890 H          1 UNK    0.0000
     29 H7          -4.4010    5.7470   -1.1760 H          1 UNK    0.0000
     30 H8          -2.2180    5.2390   -0.0330 H          1 UNK    0.0000
     31 H9           2.1600    6.8170    3.7770 H          1 UNK    0.0000
     32 H10          0.1860    7.0710    5.1480 H          1 UNK    0.0000
     33 H11         -2.0210    6.3820    4.2650 H          1 UNK    0.0000
     34 H12         -2.2630    5.7840    1.9050 H          1 UNK    0.0000
     35 H13          1.2240    4.5740   -1.4880 H          1 UNK    0.0000
     36 H14          3.2520    4.6170   -0.1620 H          1 UNK    0.0000
     37 H15          3.5650    5.3630    3.3290 H          1 UNK    0.0000
     38 H16          4.1460    6.0120    1.7960 H          1 UNK    0.0000
     39 H17          4.1027    4.2636    2.0323 H          1 UNK    0.0000
@<TRIPOS>BOND
     1    1    2 ar
     2    1   10 ar
     3    1   23 1
     4    2    3 ar
     5    2   24 1
     6    3    4 ar
     7    3   25 1
     8    4   11 ar
     9    4   26 1
    10    5    6 1
    11    5    7 ar
    12    5   11 ar
    13    6   27 1
    14    6   28 1
    15    6   29 1
    16    7    8 1
    17    7    9 ar
    18    8   18 1
    19    8   30 1
    20    9   10 ar
    21   10   11 ar
    22   12   13 ar
    23   12   17 ar
    24   12   21 ar
    25   13   14 ar
    26   13   31 1
    27   14   15 ar
```

```
    28   14   32 1
    29   15   16 ar
    30   15   33 1
    31   16   17 ar
    32   16   34 1
    33   17   18 ar
    34   18   19 ar
    35   19   20 ar
    36   19   35 1
    37   20   21 ar
    38   20   36 1
    39   21   22 1
    40   22   37 1
    41   22   38 1
    42   22   39 1
@<TRIPOS>SUBSTRUCTURE
     1 UNK      1 RESIDUE           4 A     UNK     0 ROOT
```

Section S5.2. Docking results

This section contains the tables displaying the whole solutions and clusters for each docking calculation (Tables S5.12–S5.33).

GoldScore, the scoring function used to calculate the fitness score (*F*) in this work, has been already validated for docking with metal complexes as ligands [1, 2]. The polynomial scoring function consists of a sum of four terms (Fitness score $(F) = \alpha\ S_{hb\_ext} + \beta\ S_{vdw\_ext} + \gamma\ S_{hb\_int} + \delta\ S_{int}$) accounting for hydrogen bonds between ligand and receptor (S_{hb_ext}), van del Waals interactions between ligand and receptor (S_{vdw_ext}), intramolecular hydrogen bonds of the ligand (S_{hb_int}), and a term that summarizes the intramolecular van der Waals forces of the ligand and its torsional strain energy (S_{int}). Each term is weighted by a coefficient: $\alpha = 1$, $\beta = 1.375$, $\gamma = 1$, and $\delta = 1$.

Table S5.34 shows that, concerning this study, the most important term among the four ones is S_{vdw_ext}. Regardless of its coefficient, which weighted more than the others, it is still the term that significantly contributed the most to the whole *F*. This suggest that the docking poses found in this work are dominated by the van der Waals forces between the ligand and receptor, and, more specifically, between the DNA base pairs and the aromatic moieties of the ligands (L^nH_3) and their copper complexes.

Table S5.12. Best GoldScore solutions for (*R*)-L^1H_3 with 2K4L (minor groove binding model).

**Cluster** [1]	F_{max} [2]	F_{mean} [3]	**Population** [4]
I	76.07	-	1
II	73.26	-	1
III	70.84	67.7	57
IV	67.17	65.0	32
V	66.33	64.7	7
VI	64.93	-	1
VII	63.34	-	1

[1] Clustering was performed depending on root-mean-square-deviation (RMSD) with a threshold of 2.5 Å. [2] Highest *Fitness* score in the cluster. [3] Mean *Fitness* score of the cluster. [4] Number of solutions comprised in each cluster over a total of 100 GA runs.

Table S5.13. Best GoldScore solutions for (*S*)-L^1H_3 with PDB ID 2K4L (minor groove binding model).

**Cluster** [1]	F_{max} [2]	F_{mean} [3]	**Population** [4]
I	77.72	75.0	2
II	77.39	74.0	5
III	74.52	71.8	8
IV	74.37	-	1
V	72.32	70.6	7
VI	71.22	67.1	52
VII	71.17	70.1	3
VIII	70.89	67.8	6
IX	69.24	-	1
X	68.57	66.3	10
XI	66.85	-	1
XII	64.36	-	1
XIII	64.16	64.2	2
XIV	61.09	-	1

[1] Clustering was performed depending on root-mean-square-deviation (RMSD) with a threshold of 2.5 Å. [2] Highest *Fitness* score in the cluster. [3] Mean *Fitness* score of the cluster. [4] Number of solutions comprised in each cluster over a total of 100 GA runs.

Table S5.14. Best GoldScore solutions for (*R*)-L^1H_3 with PDB ID 108D (intercalation binding model).

**Cluster** [1]	F_{max} [2]	F_{mean} [3]	**Population** [4]
I	87.98	86.3	51
II	84.31	82.1	5
III	82.87	81.4	17
IV	80.55	9.3	24
V	77.73	-	1
VI	77.18	-	1
VII	76.64	-	1

[1] Clustering was performed depending on root-mean-square-deviation (RMSD) with a threshold of 2.5 Å. [2] Highest *Fitness* score in the cluster. [3] Mean *Fitness* score of the cluster. [4] Number of solutions comprised in each cluster over a total of 100 GA runs.

Table S5.15. Best GoldScore solutions for (*S*)-L^1H_3 with PDB ID 108D (intercalation binding model).

**Cluster**[1]	F_{max}[2]	F_{mean}[3]	**Population**[4]
I	86.95	85.4	40
II	86.03	84.8	17
III	84.39	83.0	12
IV	81.87	81.1	8
V	81.29	80.6	5
VI	80.78	80.1	15
VII	79.02	-	1
VIII	77.55	77.4	2

[1] Clustering was performed depending on root-mean-square-deviation (RMSD) with a threshold of 2.5 Å. [2] Highest *Fitness* score in the cluster. [3] Mean *Fitness* score of the cluster. [4] Number of solutions comprised in each cluster over a total of 100 GA runs.

Table S5.16. Best GoldScore solutions for [Cu((*R*)-$L^1H_2^{am}$)(AcO)] with PDB ID 2K4L (intercalation binding model).

**Cluster**[1]	F_{max}[2]	F_{mean}[3]	**Population**[4]
I	72.83	69.2	83
II	71.04	69.0	11
III	70.25	69.8	2
IV	70.18	67.8	4

[1] Clustering was performed depending on root-mean-square-deviation (RMSD) with a threshold of 2.5 Å. [2] Highest *Fitness* score in the cluster. [3] Mean *Fitness* score of the cluster. [4] Number of solutions comprised in each cluster over a total of 100 GA runs.

Table S5.17. Best GoldScore solutions for [Cu((*S*)-L[1]H_2[am])(AcO)] with PDB ID 2K4L (intercalation binding model).

**Cluster** [1]	F_{max} [2]	F_{mean} [3]	**Population** [4]
I	75.86	73.1	88
II	75.72	73.9	3
III	75.67	69.0	4
IV	67.88	66.4	2
V	67.56	-	1
VI	65.93	-	1
VII	62.96	-	1

[1] Clustering was performed depending on root-mean-square-deviation (RMSD) with a threshold of 2.5 Å. [2] Highest *Fitness* score in the cluster. [3] Mean *Fitness* score of the cluster. [4] Number of solutions comprised in each cluster over a total of 100 GA runs.

Table S5.18. Best GoldScore solutions for [Cu((*R*)-L[1]H_2[am])(AcO)] with PDB ID 108D (intercalation binding model).

**Cluster** [1]	F_{max} [2]	F_{mean} [3]	**Population** [4]
I	92.35	90.2	83
II	90.30	88.3	5
III	84.89	83.8	2
IV	83.96	-	1
V	81.73	81.4	3
VI	81.04	-	1
VII	78.70	-	1
VIII	78.46		1
IX	78.36	78.3	2
X	77.27	-	1

[1] Clustering was performed depending on root-mean-square-deviation (RMSD) with a threshold of 2.5 Å. [2] Highest *Fitness* score in the cluster. [3] Mean *Fitness* score of the cluster. [4] Number of solutions comprised in each cluster over a total of 100 GA runs.

Table S5.19. Best GoldScore solutions for [Cu((*S*)-$L^1H_2^{am}$)(AcO)] with PDB ID 108D (intercalation binding model).

**Cluster** [1]	F_{max} [2]	F_{mean} [3]	**Population** [4]
I	88.18	86.0	10
II	87.60	86.0	79
III	85.95	-	1
IV	85.56	84.7	6
V	85.15	-	1
VI	85.03	84.9	2
VII	83.41	-	1

[1] Clustering was performed depending on root-mean-square-deviation (RMSD) with a threshold of 2.5 Å. [2] Highest *Fitness* score in the cluster. [3] Mean *Fitness* score of the cluster. [4] Number of solutions comprised in each cluster over a total of 100 GA runs.

Table S5.20. Best GoldScore solutions for [Cu((*R*)-$L^1H_2^{am}$)(H_2O)]$^+$ with PDB ID 2K4L (intercalation binding model).

**Cluster** [1]	F_{max} [2]	F_{mean} [3]	**Population** [4]
I	70.23	67.5	42
II	69.05	66.4	12
III	68.82	66.9	43
IV	65.01	63.3	3

[1] Clustering was performed depending on root-mean-square-deviation (RMSD) with a threshold of 2.5 Å. [2] Highest *Fitness* score in the cluster. [3] Mean *Fitness* score of the cluster. [4] Number of solutions comprised in each cluster over a total of 100 GA runs.

Table S5.21. Best GoldScore solutions for $[Cu((S)\text{-}L^1H_2^{am})(H_2O)]^+$ with PDB ID 2K4L (intercalation binding model).

**Cluster** [1]	F_{max} [2]	F_{mean} [3]	**Population** [4]
I	75.68	74.6	2
II	74.30	74.2	2
III	71.89	69.2	89
IV	70.46	70.0	2
V	70.34	68.3	2
VI	68.08	-	1
VII	60.23	-	1
VIII	59.89	-	1

[1] Clustering was performed depending on root-mean-square-deviation (RMSD) with a threshold of 2.5 Å. [2] Highest *Fitness* score in the cluster. [3] Mean *Fitness* score of the cluster. [4] Number of solutions comprised in each cluster over a total of 100 GA runs.

Table S5.22. Best GoldScore solutions for $[Cu((R)\text{-}L^1H_2^{am})(H_2O)]^+$ with PDB ID 108D (intercalation binding model).

**Cluster** [1]	F_{max} [2]	F_{mean} [3]	**Population** [4]
I	89.51	87.5	94
II	81.38	81.1	2
III	78.26	78.1	3
IV	74.80	-	1

[1] Clustering was performed depending on root-mean-square-deviation (RMSD) with a threshold of 2.5 Å. [2] Highest *Fitness* score in the cluster. [3] Mean *Fitness* score of the cluster. [4] Number of solutions comprised in each cluster over a total of 100 GA runs.

Table S5.23. Best GoldScore solutions for $[Cu((S)\text{-}L^1H_2^{am})(H_2O)]^+$ with PDB ID 108D (intercalation binding model).

**Cluster** [1]	F_{max} [2]	F_{mean} [3]	**Population** [4]
I	84.53	83.4	12
II	84.23	82.8	86
III	81.14	-	1
IV	80.38	-	1

[1] Clustering was performed depending on root-mean-square-deviation (RMSD) with a threshold of 2.5 Å. [2] Highest *Fitness* score in the cluster. [3] Mean *Fitness* score of the cluster. [4] Number of solutions comprised in each cluster over a total of 100 GA runs.

Table S5.24. Best GoldScore solutions for $[Cu((R)\text{-}L^2H_2^{am})(AcO)]$ with PDB ID 2K4L (intercalation binding model).

**Cluster** [1]	F_{max} [2]	F_{mean} [3]	**Population** [4]
I	70.25	68.1	11
II	69.69	67.2	15
III	69.60	67.7	73
IV	65.94	-	1

[1] Clustering was performed depending on root-mean-square-deviation (RMSD) with a threshold of 2.5 Å. [2] Highest *Fitness* score in the cluster. [3] Mean *Fitness* score of the cluster. [4] Number of solutions comprised in each cluster over a total of 100 GA runs.

Table S5.25. Best GoldScore solutions for [Cu((*R*)-$L^2H_2^{am}$)(AcO)] with PDB ID 108D (intercalation binding model).

**Cluster** [1]	F_{max} [2]	F_{mean} [3]	**Population** [4]
I	92.74	90.2	85
II	88.06	87.1	6
III	81.59	81.2	5
IV	79.91	79.8	2
V	78.20	-	1
VI	77.60	-	1

[1] Clustering was performed depending on root-mean-square-deviation (RMSD) with a threshold of 2.5 Å. [2] Highest *Fitness* score in the cluster. [3] Mean *Fitness* score of the cluster. [4] Number of solutions comprised in each cluster over a total of 100 GA runs.

Table S5.26. Best GoldScore solutions for [Cu((*R*)-$L^2H_2^{am}$)(H_2O)]$^+$ with PDB ID 2K4L (intercalation binding model).

**Cluster** [1]	F_{max} [2]	F_{mean} [3]	**Population** [4]
I	68.96	65.7	25
II	68.82	65.1	42
III	67.32	65.3	31
IV	67.16	-	1
V	61.91	-	1

[1] Clustering was performed depending on root-mean-square-deviation (RMSD) with a threshold of 2.5 Å. [2] Highest *Fitness* score in the cluster. [3] Mean *Fitness* score of the cluster. [4] Number of solutions comprised in each cluster over a total of 100 GA runs.

Table S5.27. Best GoldScore solutions for $[Cu((R)\text{-}L^2H_2^{am})(H_2O)]^+$ with PDB ID 108D (intercalation binding model).

**Cluster** [1]	F_{max} [2]	F_{mean} [3]	**Population** [4]
I	89.24	87.7	89
II	84.64	81.4	5
III	77.16	-	1
IV	74.62	74.5	2
V	74.12	-	1
VI	73.84	-	1
VII	72.64	-	1

[1] Clustering was performed depending on root-mean-square-deviation (RMSD) with a threshold of 2.5 Å. [2] Highest *Fitness* score in the cluster. [3] Mean *Fitness* score of the cluster. [4] Number of solutions comprised in each cluster over a total of 100 GA runs.

Table S5.28. Best GoldScore solutions for $[Cu((R)\text{-}L^3H_2^{am})(AcO)]$ with PDB ID 2K4L (intercalation binding model).

**Cluster** [1]	F_{max} [2]	F_{mean} [3]	**Population** [4]
I	74.53	70.0	66
II	73.97	-	1
III	70.58	67.1	4
IV	68.85	67.1	22
V	67.19	66.5	3
VI	66.41	-	1
VII	65.99	65.7	2
VIII	58.43	-	1

[1] Clustering was performed depending on root-mean-square-deviation (RMSD) with a threshold of 2.5 Å. [2] Highest *Fitness* score in the cluster. [3] Mean *Fitness* score of the cluster. [4] Number of solutions comprised in each cluster over a total of 100 GA runs.

Table S5.29. Best GoldScore solutions for [Cu((*R*)-$L^3H_2^{am}$)(AcO)] with PDB ID 108D (intercalation binding model).

**Cluster** [1]	F_{max} [2]	F_{mean} [3]	**Population** [4]
I	83.24	82.5	2
II	82.31	79.7	28
III	81.46	79.5	61
IV	80.46	79.0	6
V	79.26	-	1
VI	78.63	-	1
VII	74.70	-	1

[1] Clustering was performed depending on root-mean-square-deviation (RMSD) with a threshold of 2.5 Å. [2] Highest *Fitness* score in the cluster. [3] Mean *Fitness* score of the cluster. [4] Number of solutions comprised in each cluster over a total of 100 GA runs.

Table S5.30. Best GoldScore solutions for [Cu((*R*)-$L^3H_2^{am}$)(H_2O)]$^+$ with PDB ID 2K4L (intercalation binding model).

**Cluster** [1]	F_{max} [2]	F_{mean} [3]	**Population** [4]
I	68.63	66.2	9
II	66.23	62.5	17
III	65.40	62.8	25
IV	63.56	61.6	25
V	63.45	-	1
VI	63.00	61.2	20
VII	62.40	62.1	2
VIII	61.09	-	1

[1] Clustering was performed depending on root-mean-square-deviation (RMSD) with a threshold of 2.5 Å. [2] Highest *Fitness* score in the cluster. [3] Mean *Fitness* score of the cluster. [4] Number of solutions comprised in each cluster over a total of 100 GA runs.

Table S5.31. Best GoldScore solutions for [Cu((*R*)-$L^3H_2^{am}$)(H_2O)]$^+$ with PDB ID 108D (intercalation binding model).

**Cluster** [1]	F_{max} [2]	F_{mean} [3]	**Population** [4]
I	75.48	74.4	25
II	75.17	-	1
III	74.22	73.3	74

[1] Clustering was performed depending on root-mean-square-deviation (RMSD) with a threshold of 2.5 Å. [2] Highest *Fitness* score in the cluster. [3] Mean *Fitness* score of the cluster. [4] Number of solutions comprised in each cluster over a total of 100 GA runs.

Table S5.32. Best GoldScore solutions for TO with PDB ID 2K4L (intercalation binding model).

**Cluster** [1]	F_{max} [2]	F_{mean} [3]	**Population** [4]
I	71.42	69.4	53
II	70.23	67.4	11
III	70.04	68.6	14
IV	69.98	68.0	8
V	68.20	67.3	4
VI	67.28	64.2	2
VII	67.11	65.3	6
VIII	63.95	-	1
IX	61.69	-	1

[1] Clustering was performed depending on root-mean-square-deviation (RMSD) with a threshold of 2.5 Å. [2] Highest *Fitness* score in the cluster. [3] Mean *Fitness* score of the cluster. [4] Number of solutions comprised in each cluster over a total of 100 GA runs.

Table S5.33. Best GoldScore solutions for TO with PDB ID 108D (intercalation binding model).

**Cluster** [1]	F_{max} [2]	F_{mean} [3]	**Population** [4]
I	88.13	86.6	5
II	87.76	86.0	67
III	87.29	86.5	27
IV	85.85	-	1

[1] Clustering was performed depending on root-mean-square-deviation (RMSD) with a threshold of 2.5 Å. [2] Highest *Fitness* score in the cluster. [3] Mean *Fitness* score of the cluster. [4] Number of solutions comprised in each cluster over a total of 100 GA runs.

Table S5.34. *Fitness* score analysis of the best poses for each docking calculation.

**Ligand**	**Binding mode (PDB ID)**	***F***	S_{hb_ext}	S_{vdw_ext} [1]	S_{hb_int}	S_{int}
(*R*)-$L^1H_3^{am}$	Groove (2K4L)	76.1	6.09	79.17	0.00	-9.21
	Intercalation (108D)	88.0	0.00	88.29	0.00	-0.31
(*S*)-$L^1H_3^{am}$	Groove (2K4L)	77.7	6.30	71.93	0.00	-0.51
	Intercalation (108D)	87.0	1.72	89.53	0.00	-4.29
[Cu((*R*)-$L^1H_2^{am}$)(AcO)]	Groove (2K4L)	72.8	7.13	73.36	0.00	-7.66
	Intercalation (108D)	92.4	1.70	90.98	0.00	-0.34
[Cu((*S*)-$L^1H_2^{am}$)(AcO)]	Groove (2K4L)	75.9	6.60	71.68	0.00	-2.41
	Intercalation (108D)	88.2	4.62	86.27	0.00	-2.71
[Cu((*R*)-$L^1H_2^{am}$)(H_2O)]$^+$	Groove (2K4L)	70.2	0.22	72.26	0.00	-2.23
	Intercalation (108D)	89.5	0.00	89.69	0.00	-0.18
[Cu((*S*)-$L^1H_2^{am}$)(H_2O)]$^+$	Groove (2K4L)	75.1	2.68	74.53	0.00	-2.15
	Intercalation (108D)	84.5	0.00	87.67	0.00	-3.13
[Cu((*R*)-$L^2H_2^{am}$)(AcO)]	Groove (2K4L)	70.3	0.11	71.97	0.00	-1.82
	Intercalation (108D)	92.7	1.35	92.03	0.00	-0.64
[Cu((*R*)-$L^2H_2^{am}$)(H_2O)]$^+$	Groove (2K4L)	69.0	3.50	67.65	0.00	-2.19
	Intercalation (108D)	89.2	0.00	89.51	0.00	-0.27
[Cu((*R*)-L^3H_2-κS^{am})(AcO)]	Groove (2K4L)	74.5	1.75	74.69	0.00	-1.91
	Intercalation (108D)	83.2	0.44	83.44	0.00	-0.63
[Cu((*R*)-L^3H_2-κS^{am})(H_2O)]$^+$	Groove (2K4L)	68.6	8.80	62.85	0.00	-3.02
	Intercalation (108D)	75.5	1.97	79.96	0.00	-6.44
TO	Groove (2K4L)	71.4	0.00	73.11	0.00	-1.69
	Intercalation (108D)	88.1	0.00	90.94	0.00	-2.81

[1] This term is multiplied by the coefficient β = 1.375.

Section S6. References

1. Sciortino, G.; Sanna, D.; Ugone, V.; Lledós, A.; Maréchal, J.-D.; Garribba, E., Decoding Surface Interaction of $V^{IV}O$ Metallodrug Candidates with Lysozyme. *Inorg. Chem.* **2018,** 57, 4456-4469.
2. Sciortino, G.; Sanna, D.; Ugone, V.; Micera, G.; Lledós, A.; Maréchal, J.-D.; Garribba, E., Elucidation of Binding Site and Chiral Specificity of Oxidovanadium Drugs with Lysozyme through Theoretical Calculations. *Inorg. Chem.* **2017,** 56, 12938-12951.
